# Spider wrapping silk fibre architecture arising from its modular soluble protein precursor

**DOI:** 10.1038/srep11502

**Published:** 2015-06-26

**Authors:** Marie-Laurence Tremblay, Lingling Xu, Thierry Lefèvre, Muzaddid Sarker, Kathleen E. Orrell, Jérémie Leclerc, Qing Meng, Michel Pézolet, Michèle Auger, Xiang-Qin Liu, Jan K. Rainey

**Affiliations:** 1 Department of Biochemistry & Molecular Biology, Dalhousie University, Halifax, NS, Canada; 2 Institute of Biological Sciences and Biotechnology, Donghua University, Shanghai, P.R. China; 3 Département de Chimie, Regroupement québécois de recherche sur la fonction, la structure et l'ingénierie des protéines (PROTEO), Centre de recherche sur les matériaux avancés (CERMA) Université Laval, Québec, QC, Canada; 4 Department of Chemistry, Dalhousie University, Halifax, NS, Canada

## Abstract

Spiders store spidroins in their silk glands as high concentration aqueous solutions, spinning these dopes into fibres with outstanding mechanical properties. Aciniform (or wrapping) silk is the toughest spider silk and is devoid of the short amino acid sequence motifs characteristic of the other spidroins. Using solution-state NMR spectroscopy, we demonstrate that the 200 amino acid *Argiope trifasciata* AcSp1 repeat unit contrasts with previously characterized spidroins, adopting a globular 5-helix bundle flanked by intrinsically disordered N- and C-terminal tails. Split-intein-mediated segmental NMR-active isotope-enrichment allowed unambiguous demonstration of modular and malleable “beads-on-a-string” concatemeric behaviour. Concatemers form fibres upon manual drawing with silk-like morphology and mechanical properties, alongside secondary structuring and orientation consistent with native AcSp1 fibres. AcSp1 structural stability varies locally, with the fifth helix denaturing most readily. The structural transition of aciniform spidroin from a mostly α-helical dope to a mixed α-helix/β-sheet-containing fibre can be directly related to spidroin architecture and stability.

Spiders can produce up to seven types of silk that surpass synthetic materials in ultimate tensile strength (i.e., maximum stress, or force per cross-sectional area, withstood before breaking) and toughness (i.e., energy absorbed before breaking) per unit weight^1^,^2^,^3^. Spider silk proteins, or spidroins, are large (250–500 kDa) and have a general architecture comprising a repetitive domain, accounting for at least 90% of the total protein sequence, flanked by non-repetitive N- and C-terminal domains. Spidroins are highly soluble in the gland and, when needed, efficiently self-assemble into insoluble fibres^4^. The protein secondary structure also changes during this process, typically from a soluble mixture of random-coil, polyproline-II- helices and/or α-helices to a fibre enriched in β-sheet content but still exhibiting significant disorder^5^,^6^,^7^.

Aciniform silk is the toughest spider silk and is composed of the protein aciniform spidroin 1 (AcSp1)^8^. It is the primary component of wrapping silk, which is used to wrap and immobilize prey. Present knowledge of spider silk structure and function is heavily based on dragline silk, the strongest of the spider silks^9^. During the transition from the soluble state to the fibre form, dragline silk converts from a disordered state^10^, likely exhibiting polyproline-II and transient α-helical character^7^,^11^, to a β-sheet microcrystal-rich aggregate^12^,^13^. AcSp1 from *Nephila clavipes*, conversely, is ~50% α-helical in the aciniform gland and ~24% α-helical and ~30% β-sheet in the solid fibre^7^. Retention of significant α-helical content in the insoluble form is unique to the aciniform and piriform silks, with piriform silk morphology differing in that it functions in disc form rather than as an isolated fibre^7^,^14^,^15^.

A typical hallmark of spidroins is the presence of small (usually ≤10 amino acid) primary structural motifs (GGX, GPGXX, A_n_, etc.)^1^,^16^,^17^. These motifs have been directly linked to specific mechanical properties, particularly for dragline silk^4^,^13^,^18^. In contrast to this, AcSp1 is composed of concatenated ~200–400 amino acid repeat units completely lacking these short motifs^8^. AcSp1 primary and secondary structure as well as fibre mechanical properties therefore differ from the other spidroins and the link between these characteristics remains elusive.

To date, only three spidroin repetitive domain structures have been solved^19^,^20^ alongside several non-repetitive N- and C-terminal domain structures^20^,^21^,^22^,^23^,^24^,^25^,^26^. The reported repetitive domain structures are all highly similar seven-helix bundles^19^,^20^. Two of these are of tubuliform spidroin TuSp1 repeat units 1 and 2^20^ and the third is of a putative AcSp1 repeat unit^19^, all recombinant proteins based upon genes annotated from *Nephila antipodiana.* Tubuliform (or cylindriform) spidroin is quite divergent from the other spidroin family members, with a particularly low glycine and elevated serine content. Unlike aciniform spidroin, tubuliform spidroin has also been shown to undergo a complete conversion to β-sheet/random-coil in the fibre without retention of α-helical character^7^. The previously reported structural similarity between AcSp1 and TuSp1 is therefore unexpected.

Here, we use solution-state NMR spectroscopy to determine the atomic-level structure and dynamics of recombinant AcSp1 based upon the *Argiope trifasciata* spidroin. In the native form, this AcSp1 protein is a concatemer of a 200 amino acid repeat unit (referred to as the W unit herein) iterated at least 14 times and flanked by non-repetitive C-terminal and, putatively, N-terminal domains^8^,^27^. We demonstrate the AcSp1 structure to be unlike the previously determined spidroin repeat unit structures and, in addition, validate and present the AcSp1 repeat domain in the structural context of the concatemer. Fibres may be readily drawn from our concatemer NMR samples, with morphology and secondary structure properties highly similar to native AcSp1 fibres from *Argiope aurantia* and mechanical properties approaching those of native silk.

## Results

### The Structure of W_1_

**Figure 1:**
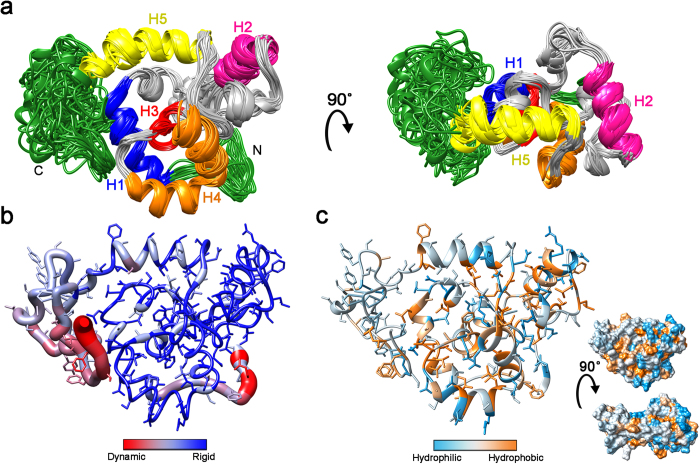
Solution-state NMR structure of W1. (**a**) Overlay of 20 lowest energy members of the NMR ensemble. Each helix in the converged domain is coloured differently, as annotated directly on the figure; the intrinsically disordered portions excluded from r.m.s.d. calculations are in green. (**b**) Heteronuclear ^1^H -^15^N NOE enhancement factors represented on the W_1_ lowest energy structure (bar graph in Fig. 2c). (**c**) The lowest energy structure coloured according to the Kyte-Doolittle hydrophobicity scale^72^ shown in ribbon/stick and surface (inset) representations.

**Figure 2:**
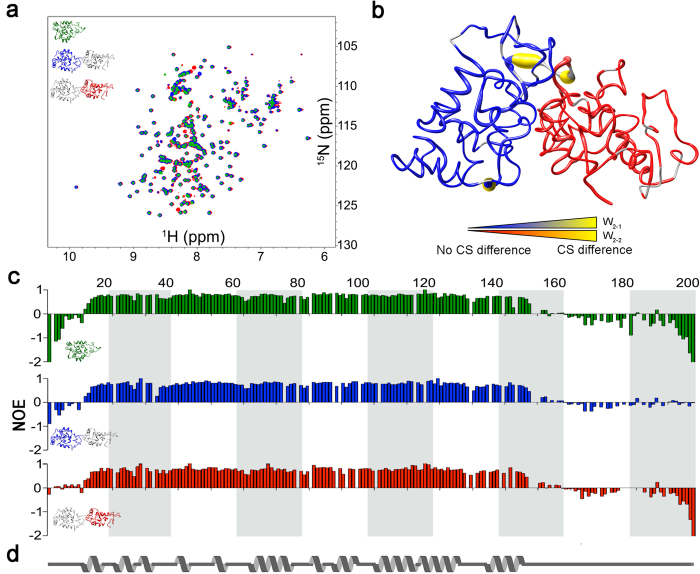
Modularity of the repeat unit of AcSp1. (**a**) Overlay of ^1^H-^15^N HSQC experiments for W_1_ (green) and W_2_ concatemers with first (blue; W_2−1_) or second (red; W_2−2_) W unit ^15^N-enriched. (**b**) Combined chemical shift difference between W_2_ and W_1_ (W_2−1_ blue; W_2−2_ red; data are presented in Supplementary Fig. S6). (**c**) Heteronuclear ^1^H-^15^N NOE enhancement factors for W_1_ (green), W_2−1_ (blue), and W_2−2_ (red). (**d**) Schematic of secondary structure as a function of sequence based upon DSSP^63^ secondary structure assessment of the 20-member ensemble in Fig. 1.

The soluble form of the W unit (W_1_: 199 amino acids, lacking the C-terminal serine of the 200 amino acid repeat) exhibits a well-folded and tightly packed ellipsoidally-shaped helical core over residues 12–149 flanked by unstructured tails (Fig. 1). Heteronuclear ^1^H-^15^N nuclear Overhauser effect (NOE) enhancement factors^28^ are indicative of a rigid protein core, reflected by positive enhancement factors, and flexible dynamic tails, reflected by negative or near-zero enhancement factors (Figs 1b and 2c), corroborating the localization of folded vs. disordered domains^29^,^30^. The disordered tails of W_1_ are also evident both from a lack of inter-residue ^1^H-^1^H NOE restraints^31^ and according to chemical shift-derived TALOS+ dihedral angle assignments^32^ (Supplementary Figs. S1a and S2). The solution-state W_1_ structure therefore comprises a compact, predominantly helical globular core with intrinsically disordered N- and C-terminal tails.

Although chemical shift patterns are indicative of ~6 α-helices^33^, our structural refinement (Table 1) using ^1^H-^1^H NOE-based distance restraints with >20 NOE contacts per residue over most of the globular region (Supplementary Fig. S1), coupled with TALOS+^32^ dihedral angle restraints (Supplementary Figs. S2 and S3) and hydrogen-deuterium exchange-derived hydrogen bond restraints^34^ (Supplementary Figs. S1b and S3), demonstrates 5 α-helical regions. Despite the lack of a continuous helix in the putative helix 2 of the original topology^33^, ~65% of residues in the S40-G60 stretch adopt α-helical ϕ and ψ dihedral angles (Supplementary Fig. S4). The observation of chemical shifts implying α-helical structure is, therefore, not unreasonable. As a whole, this segment of W_1_ is well converged and structured, with localized helical, β- and γ-turns located in the core of the protein. Also supporting this non-canonical structuring, the N-terminal region of this segment includes several residues exhibiting chemical shifts outside the statistical norm^33^. Further evidence for a five-helix structural topology is clear both on the basis of regions exhibiting expected^31^ α-helical NOE connection patterns (Supplementary Fig. S3) and in direct analysis of the final ensemble of NMR structures. Also of note, the two phenylalanines in the central helix (F90 and F95) are mildly solvent exposed on opposing sides of W_1_ while the other 8 aromatic amino acids fall in the top 25% most solvent exposed residues over the NMR ensemble (Supplementary Fig. S5). Exposure of aromatic moieties in this manner may promote protein-protein association during fibre self-assembly.

### Effect of Concatenation upon the W Unit

**Figure 3:**
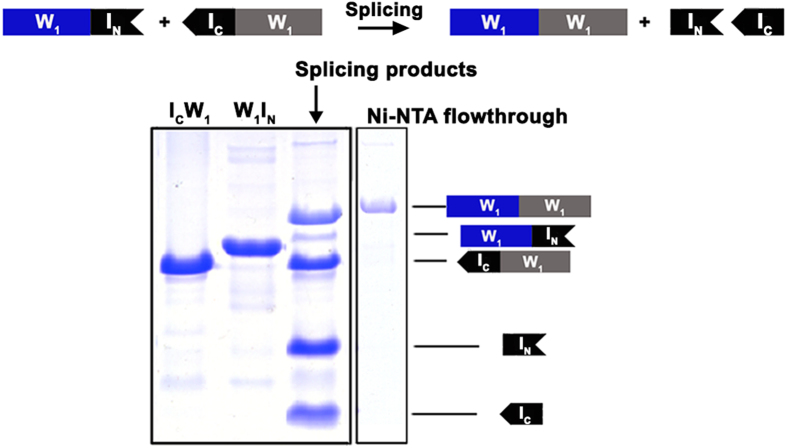
Segmental labelling of W_2_ by split-intein-mediated *trans*-splicing (I_N_ and I_C_ refer to N- and C-terminal intein fragments, respectively). In the illustrated splicing scheme, the W_1_I_n_ fusio_n_ protein is enriched with NMR active isotopes (blue), while the I_c_W_1_ fusion protein is at natural abundance (grey). In the illustrated SDS-PAGE gel, splicing products were passed through a Ni-NTA column, allowing the spliced W_2_ to be collected in the flow-through while all other proteins were His_6_-tagged and thus retained in the column.

To investigate the effect of concatenation of the W unit upon protein structuring and dynamics by NMR, concatemers of two W units (W_2_) were studied. To unambiguously distinguish each W unit, selective enrichment of one of the two W units with NMR-active ^13^C- and/or ^15^N-isotopes was carried out using split-intein-mediated *trans*-splicing^35^,^36^. This was feasible by separately expressing fusion proteins of one W unit with the appropriate *Ssp* GyrB split-intein^37^ fragment in *E. coli* and *trans*-splicing the two W units together to produce a concatemer linked by a native peptide bond (Fig. 3).

^1^H-^15^N HSQC experiments for W_1_ and for each W unit in W_2_ demonstrate strikingly similar chemical shift patterns (Figs 2a,b and Supplementary Fig. S6). Quantitative backbone chemical shift comparison^38^ provides further insight, clearly demonstrating that only the residues immediately adjacent to the covalent link between W units differ between W_1_ and W_2_ (Fig. 2b and Supplementary Fig. S6a). Echoing this behaviour, W unit independence likely extends to the 3-unit concatemer (W_3_), as the 1D ^1^H-NMR spectra of W_1_, W_2_ and W_3_ are practically indistinguishable with exception of intensity, which increases in direct proportion to the number of W units present in the concatemer (Supplementary Fig. S6b).

The steady-state heteronuclear ^1^H-^15^N NOE enhancement factor provides a position-specific probe^39^ of intramolecular dynamics because it is highly sensitive to the local effective correlation time^28^,^40^. Amide H-N bonds exhibiting a heteronuclear NOE enhancement factor of 0.65 or greater are typically attributed to regions of the protein that experience minimal internal motion faster than the ~5–20 ns rotational correlation time typical of a protein^41^, although it should be noted that the exact value of enhancement factor used as a cut-off to identify internal motion depends upon the system in question^42^. Comparison of heteronuclear ^1^H-^15^N NOE enhancement factors as a function of backbone position in each W unit of W_2_ relative to W_1_ demonstrates that, in all cases, the helical domain exhibits high enhancement factors typical of tumbling as a globular protein domain while the first 11 and last 50 residues of each W unit display decreased enhancement factors typical of internal motion on the ps-ns time scale (Fig. 2c). Logically, concatenation produces some apparent damping of the dynamics in W_2_ at the link between units vs. at the termini. In all cases, the N- and C-terminal portions of the repeat domain retain the elevated dynamics expected of an intrinsically disordered segment^30^.

### Hydrodynamics of AcSp1

To better characterize AcSp1 architecture, translational diffusion coefficient (D_C_) values for W_1_, W_2_ and W_3_ were determined using pulsed field gradient diffusion-ordered NMR spectroscopy (DOSY)^43^. For comparison, dynamic light scattering (DLS) was used to determine the hydrodynamic diameter (d_H_) of each W species. The observed D_C_ of W_1_ was in good agreement with the NMR-derived structural ensemble (Supplementary Table S1). No component to the scattering decay curve attributable to a monomeric species was observed for filtered W_1_ in acetate buffer by DLS, where the predominant species giving rise to scattering appeared to be nanoparticles of ~100–200 nm size consistent with our previous DLS studies of W_1_ in phosphate buffer^44^. The source of this discrepancy between NMR (where a 100–200 nm particle would be unobservable by standard solution-state methodology) and DLS (where only the large species is observed) remains elusive. W_2_ and W_3_, conversely, exhibited clear scattering from monomeric species. The d_H_ for W_2_ agrees outstandingly well with that inferred from the viscosity-corrected^45^ D_C_ while the d_H_ of W_3_ is inferred to be slightly more compact by DLS than by DOSY (Supplementary Table S1). As a whole, the hydrodynamic properties of W_2_ and W_3_ are highly consistent between DOSY and DLS, while those of W_1_ agree between DOSY and the high-resolution structural ensemble.

### Concatemeric Structural Ensembles

**Figure 4:**
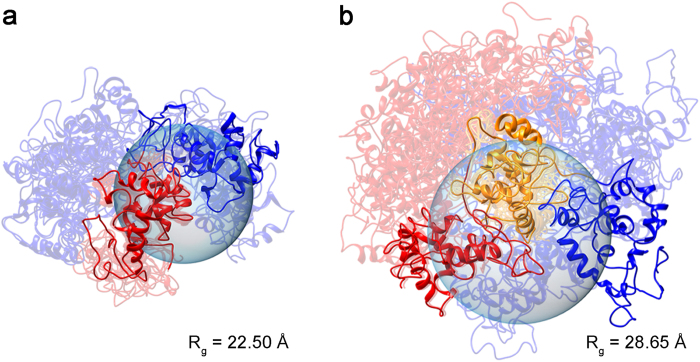
Structural ensembles (semi-transparent colouring) underlying the representative structure (solid colouring; Rg closest to that determined by DOSY NMR) of W2 (**a**) and W_3_ (**b**). Ensembles (10 members shown) were calculated using concatenated sets of W_1_ NMR restraints with an R_g_ restraint estimated from the observed D_C_^DOSY^.

DOSY-derived D_C_ values were used to infer^46^ a radius-of gyration (R_g_) for each AcSp1 species (Supplementary Table S1). Calculation of a W_1_ structural ensemble was carried out with addition of an R_g_ restraint^47^, using restraint weighting appropriate to ensure that other NMR-derived restraints were not violated. The incorporation of R_g_ as a restraint for W_1_ led to no significant change in the averaged hydrodynamic properties of the structural ensemble (Supplementary Table S1). Based upon the outstanding agreement between W_1_ and W_2_ backbone chemical shifts (Fig. 2), W_2_ and W_3_ structural ensembles (Fig. 4 and Supplementary Table S1) were calculated using concatenated sets of the W_1_ NMR restraints (Table 1). Unlike the observation with W_1_, the levels of agreement between the measured D_C_ and that inferred from structural ensembles for W_2_ and W_3_ improved with addition R_g_ restraints^47^ (Supplementary Table S1) without increased violation of the other NMR-derived restraints. The W_2_ and W_3_ structural ensembles calculated incorporating R_g_ showed a general increase in compactness in comparison to those without.

### Properties of W_2_ Fibres

**Figure 5:**
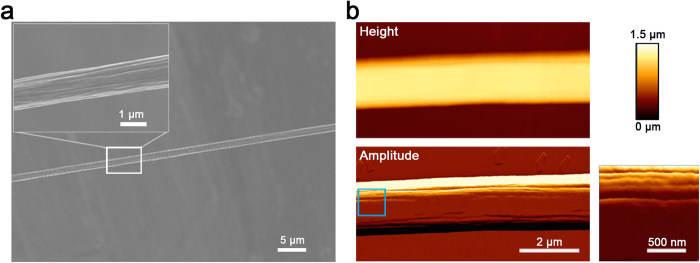
W_2_ fibre surface observed by SEM (**a**) and intermittent-contact AFM (**b** – colour scale for height image shown; boxed region of amplitude image shown at higher resolution to the right).

The functional relevance of the NMR conditions for fibre formation was tested based on our previous demonstration that silk-like fibres can be manually drawn from solutions of concatemers comprising 2 to 4 W units^48^. Fibres with ~1.5 μm diameter and a surface morphology of smaller fibrils aligned parallel to the fibre long axis (Fig. 5), consistent with our previous recombinant W fibre characterization, could be readily drawn from NMR samples of W_2_ proteins. Interestingly, the strength and toughness of recombinant W fibres appears approximately proportional to concatemer size (Table 2), with W_2_ (whether produced intact or intein-spliced) exhibiting values ~half those of W_4_ fibres^48^ and ~5–10% those of native *A. trifasciata* aciniform silk (W_14_ or larger, plus non-repetitive domain(s)^8^).

**Figure 6:**
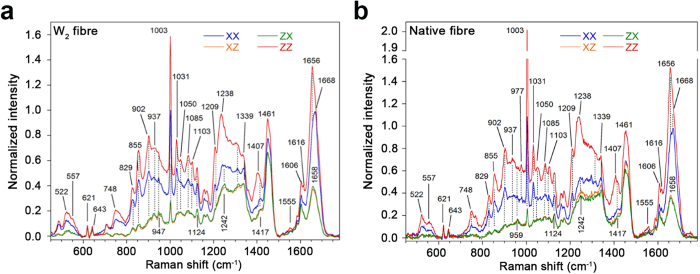
Comparative secondary structuring and orientation observed by polarized Raman spectromicroscopy for the indicated fibre type.

Comparison of polarized Raman spectromicroscopy of W_2_ fibres and of natural aciniform silk fibres from *Argiope aurantia* demonstrates very close agreement in amino acid composition, secondary structure and molecular orientation (Fig. 6). Curve-fitting of the amide I band of the orientation-insensitive spectra^11^ demonstrates a significant decrease in α-helicity and appearance of β-sheet character in W_2_ fibres relative to the solution-state structure, highly consistent with wrapping silk fibres produced by both *A. aurantia* (decomposition in Supplementary Fig. S7) and *N. clavipes*^7^ (Table 3). Analysis of relative amide I and amide III band peak height in the XX and ZZ polarized Raman spectra demonstrates that the α-helices and β-sheets are predominantly aligned along the fibre axis.

### Local Stability Variation Within the W unit

Fibre formation by W_2_ is inhibited by the addition of the chaotropic reagents urea and guanidinium chloride (GdmCl) or the zwitterionic detergent dodecylphosphocholine (DPC) above its critical micelle concentration (CMC ≈ 1.1 mM^49^). Far-UV circular dichroism (CD) spectroscopy demonstrates complete denaturation of W_1_ and W_2_ upon titration with both chaotropes (Supplementary Figs. S8a and b), whereas DPC-induced changes are subtle at the global level reflected by CD spectroscopy (Supplementary Figs. S8c and d) but drastic at the backbone amide level observed by NMR spectroscopy once the CMC is exceeded (Supplementary Figs. S9 and S10). To rule out effects of ionic strength vs. the chaotrope activity of GdmCl, a control titration was carried out for W_1_ using NaCl (Supplementary Fig. S11). Ionic strength-dependent chemical shift perturbation is clearly apparent, but the typical W_1_ HSQC spectral pattern is maintained with NaCl, unlike with GdmCl, where a contraction in cross-peak dispersion consistent with denaturation is observed.

**Figure 7:**
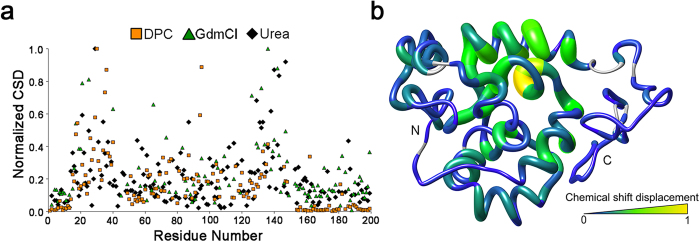
Site-specific perturbation of W_1_ upon denaturation or detergent treatment. (**a**) Normalized combined chemical shift displacement (CSD)^38^ as a function of amino acid position in W_1_ for titrations with the detergent dodecylphosphocholine (DPC; 20 mM endpoint) or the indicated chaotropic denaturant (2.5 M urea or 0.8 M GdmCl titration points). (**b**) Representation by thickness and colour of average CSD caused by urea, GdmCl and DPC on a cartoon representation of the lowest-energy member of the W_1_ structural ensemble.

In each of the chaotrope and DPC titrations, helix 5 (residues 135–149, yellow in Fig. 1a) and the residues located directly underneath it are the most readily perturbed portions of the W unit (Fig. 7 and Supplementary Fig. S9). Correspondingly, of all helical segments in W_1_, helix 5 was the least protected from H/D exchange in buffer (Supplementary Fig. S1b). In converse to the perturbation at helix 5, even at the DPC endpoint, the W_2_ linker region remained unperturbed (Supplementary Fig. S10). The propensity of the fifth helix towards unfolding may be rooted in its primary structure, given that a variety of sequence-based analysis algorithms predict the entire stretch of W_1_ over residues 125–199, including helix 5, to be intrinsically disordered (Supplementary Fig. S2).

## Discussion

The mechanical properties of spider silks are predominantly linked to the spidroin repetitive domain. Comparison of repetitive domain structuring before and after fibre formation is fundamental to understanding both the exceptional mechanical properties of spider silks and their self-assembly. We present the structure of the repeat unit of the toughest spider silk, aciniform silk, from *A. trifasciata*. The observed globular 5-helix W_1_ architecture has no structural resemblance (Supplementary Fig. S12) to the 7-helix bundle previously reported for tubuliform spidroin repetitive unit and for a truncated, putative aciniform spidroin repetitive unit. These latter two spidroin repetitive units were both identified from an expressed sequence tag library from *N. antipodiana* and share only ~27% and 22% pairwise sequence identity with W_1_, respectively^19^. W unit structuring generally appears insensitive to conditions with robust refolding following thermal denaturation^44^; therefore, the observed difference in fold between W unit and the other spidroin repetitive units seems unlikely to be caused by differences in experimental conditions. Rather, this appears to be a fundamental architectural difference with as yet unknown source.

In each of the previous studies, only isolated repeats were employed and the impact of repeat unit concatenation was not investigated at the atomic level or discussed. Chemical shift and backbone amide dynamics comparison, alongside hydrodynamics characterization, allowed us to demonstrate that the repeat units in W_1_ and W_2_ are structurally indistinguishable and that there are no detectable persistent interactions between the two repeat units in W_2_. Modularity of behaviour also extends to W_3_, implying that the W_1_ structure is representative of the repeat unit structure in the context of a large, multiple-repeat protein. For the first time, we demonstrate the modularity of a spidroin repetitive domain at the atomic level. As a whole, the W_1_ unit is a highly tractable module for study, providing direct insight into much larger proteins otherwise infeasible to study at atomic resolution.

Notably, the conditions under which we have characterized W_2_ are clearly directly relevant to fibre formation given that fibres formed by manual pulling, including directly from NMR buffer, have architecture and orientation of structural units consistent with natural wrapping silk from *A. aurantia.* Fibres, conversely, cannot be pulled from solutions of W_1_ alone^48^. Structural study of the functional W_2_ fibre precursor, consisting of identical W_1_ 19 kDa subunits (with ~50% of content being Gly, Ala and Ser) concatenated into a repetitive 38 kDa protein was possible only through intein-mediated protein *trans*-splicing. As highlighted in recent literature reviews^50^,^51^,^52^, such a strategy has strong potential for direct characterization of other comparably challenging and interesting systems. The similarity in both structure and dynamics of W_1_ and W_2_ implies that W_1_ is directly representative of the functional AcSp1 repeat unit in the state where it is primed for fibre formation.

Concatemers larger than W_2_ tend to oligomerize in solution, with faster rates observed for larger concatemers, precluding extended solution-state NMR studies. W_4_, for example, oligomerized into a visible precipitate during DOSY experiments, rendering NMR-based hydrodynamics characterization infeasible. Structural studies of tubuliform spidroin TuSp1 domains were performed in DPC micelles, as these conditions stabilized the monomeric forms of a variety of TuSp1 constructs^20^. We had, therefore, hoped that DPC would act to stabilize large W unit concatemers sufficiently for hydrodynamics characterization. Unexpectedly, however, we observed that DPC specifically inhibited fibre formation of W_2_ despite minimal structural perturbation evident by CD spectroscopy. This observation, in light of similar inhibition of fibre-formation behaviour upon addition of urea and GdmCl, spurred our investigation of the effects of DPC titration upon W unit structuring. Fortuitously, the NMR spectroscopic behaviour of the W unit in the presence of DPC allowed for much clearer tracking of individual HSQC-based N-H correlations than in chaotrope solutions, allowing unambiguous demonstration of localized perturbation in both W_1_ and W_2_.

The observation of a locally destabilized region of the W unit folded domain implies potential for localized structural modulation during fibre formation. Structural transition could be initiated by application of shear forces, for example, during the manual fibre pulling process. Helix 5, which is positioned on the surface of the helical bundle without extensive inter-helical interactions, normally positions the globular bead-like domain proximally to the linker (Fig. 1). Its denaturation and concomitant extension would therefore allow for structural decompaction through decreased constraint upon the linker. Protein-protein interaction would, in turn, be facilitated through an overall increase in surface area and introduction of a longer and more flexible string-like linker with potential to entangle with proximal molecules. Future testing of both the effects of stabilizing this helix (e.g. through replacement with a stabilized helix, as employed probing β_2_-microglobulin amyloid formation^53^) and of holding the helix in place (e.g., with a disulfide, as employed to trap the SH3 domain transition state^54^) would be ideal to provide a more detailed characterization of the role of localized unfolding in aciniform silk fibre formation.

Notably, the W_2_ fibre demonstrates a secondary structural transformation from its soluble precursor very similar to that of native wrapping silk of *N. clavipes.* Although the dope has not been characterized in the *A. aurantia* aciniform gland, the W_2_ fibre also exhibits outstanding agreement with *A. aurantia* wrapping silk fibre structural properties. In short, partial α-helix to β-sheet transition is observed in all cases upon conversion from the soluble spidroin to fibrous silk state. Chemical shift-based secondary structure propensity (SSP) analysis^55^ implies that the only regions of W_2_ with nascent β-strand propensity in the soluble state are found flanking helix 5 and proximal to the junction between W units (Supplementary Fig. S10c). It is therefore probable that β-Sheet formation is seeded on the helix 5 face of the W unit and within the linker between neighbouring W units. A significant portion of the linker, however, likely retains its disordered state given that the final proportion of disorder in the fibre is ~40%. The fact that 6 of 8 prolines are found in a 20-residue stretch of the linker (between residue numbers 172 and 191 of the W unit) provides a further, significant constraint against complete conversion of the disordered linker to β-sheet.

Based upon these findings, fibrillogenesis may be hypothesized to take place as follows. First, localized unfolding of helix 5, encouraged by shear forces, decompacts a given W unit, inducing increased intermolecular interactions. Intermolecular β-sheet formation subsequently occurs, perhaps following alleviation of some mechanical force. The resulting fibre consists of helical domains composed of the stable helix 1–4 core of the bead-like globular portion of AcSp1 alongside β-sheet-rich domains. A significant proportion of disorder, or non-canonical secondary structuring, would arise in the linker and, potentially, the structured but non-helical segment between helices 1 and 2 (residues 40–60 of W_1_), providing elasticity to the fibre. The end-result would be a fibre composed of a mixture of discrete α-helical and β-sheet domains embedded in a disordered and elastic protein milieu.

In summary, the soluble form of the repetitive domain of the spider wrapping silk protein AcSp1 is composed of globular domains (“beads”) containing 5 helical segments held together by compact but intrinsically disordered linkers (“strings”). This beads-on-a-string architecture was shown unambiguously by independently and selectively enriching each W unit in the two-unit concatemer (W_2_) with NMR-active isotopes using split-intein-mediated *trans*-splicing. Comparison of Raman spectra of fibres drawn directly from recombinantly produced W_2_ protein samples and of natural *A. aurantia* aciniform silk fibres demonstrate strong similarity in amino acid composition, secondary structure and molecular orientation. A fibrous wrapping silk architecture composed of discrete oriented α-helix and β-sheet rich modules in a setting of intrinsically disordered protein would logically arise from the observed locally variable helical stability in the soluble form of the AcSp1 repeat unit. The mixture of α-helical, β-sheet and non-canonical secondary structures making up the outstandingly tough spider wrapping silk fibre thus can be related directly to the modular architecture and properties of its soluble precursor protein.

## Methods

### Protein Expression and Labelling

SUMO-W_1_ and -W_2_ fusion proteins were constructed as contiguous genes in a modified pET32 plasmid, expressed, labelled with NMR active isotopes, cleaved using SUMO protease and reverse purified as previously described^33^,^48^. For segmental-labelling, two constructs containing the N-precursor (W_1_I_N_: W_1_ + intein N-fragment (I_N_) + His_6_ tag) and C-precursor (I_C_W_1_: His_6_ tag + intein C-fragment (I_C_) + W_1_; this fusion protein required addition of urea for solubilisation at all stages from lysis (4 M) through to purification (2 M)) were constructed for use with the split-intein *Ssp* GyrB^37^. N- and C-precursors were purified by Ni-NTA affinity chromatography. To segmentally-label W_2_ proteins through intein *trans*-splicing, excess of the unlabelled N- or C-precursor was mixed with the corresponding isotope-enriched C- or N- precursor, respectively, to make efficient use of isotope enrichment. The splicing reaction was carried out in purification elution buffer (50 mM sodium phosphate, 300 mM NaCl, 250 mM imidazole, pH 8.0) with 1 mM DTT at 4 **°**C for > 6 hours. The mixture was then dialyzed against 50 mM potassium phosphate, pH 7.5 at 4 °C for > 2 hours and reverse purified by passing through Ni-NTA Sepharose. Any remaining unreacted precursors and all intein fragments had His_6_ tags and were thus trapped, leaving the tag free W_2_ protein to flow through the column. Splicing and purification efficiencies were analysed by SDS-PAGE and visualized by staining with Coomassie Brilliant Blue R-250.

### NMR Spectroscopy

W_1_ (~0.2 mM) NMR experiments in NMR buffer (20 mM sodium acetate, 1 mM 2,2-dimethyl-2-sila-pentane-5-sulfonic acid (DSS), 1 mM NaN_3_; pH 5) in H_2_O:D_2_O at 9:1 (v:v) were acquired, processed and assigned as previously described^33^ with the addition of an aromatic ^13^C-edited NOESY-HSQC (mixing time: 85 ms). Backbone NMR experiments were acquired for ^13^C/^15^N enriched segmentally-labelled W_2_ proteins (~0.2–0.3 mM in NMR buffer) using a 16.4 T Avance III spectrometer equipped with a 5 mm TCI cryoprobe (Bruker, Milton, ON, Canada) at 303.15 K in the same manner as for W_1_. ^1^H-^15^N HSQC experiments were used to monitor H/D exchange at 0 h (control), 6 h and 40 h time point at 16.4 T with 24 scans, 2048 and 192 points in the ^1^H and ^15^N dimensions respectively, and a recovery delay of 1.5 s. H/D exchange was performed by replacement of the original NMR buffer in 90% H_2_O and 10% D_2_O with NMR buffer in 100% D_2_O using centrifugal 15 mL spin dialysis filters (EMD Millipore, Billerica, MA). Heteronuclear NOE enhancement factors were measured using the standard Bruker sensitivity enhanced ^1^H-^15^N HSQC experiment with saturation during the recycle delay performed in an interleaved manner for recording relative NOE enhanced vs. unenhanced signal intensity (hscqnoef3gpsi3d; d1 of 5 s). Enhancement factors are reported as I(sat)/I(ref), where I(sat) is a given peak height under saturation and I(ref) the corresponding height without. Experimental data were processed with NMRPipe^56^ and assigned via CcpNmr Analysis^57^. Combined chemical shift displacements^38^ for each residue in each W unit in W_2_ relative to W_1_ were calculated using the backbone H, N, CA, and CO chemical shifts weighted by gyromagnetic ratio.

### W_1_ Restraint Refinement and Structure Calculation

NOE-derived distance restraints for W_1_ were assigned in CcpNmr Analysis^57^ for the following spectra: a ^15^N-edited NOESY-HSQC, a ^1^H-^13^C HSQC-NOESY-^1^H-^15^N HSQC, a ^13^C-edited NOESY-HSQC, and an aromatic ^13^C-edited NOESY-HSQC. Dihedral angle restraints were produced using TALOS+^32^. H-bond restraints were generated on the basis of assigned ^1^H-^15^N HSQC peaks remaining following 6 h of H/D exchange based on the premise that backbone amide peaks resistant to exchange are located in helical regions^34^. Highly ambiguous NOEs were filtered with ARIA 2.1^58^, using NOE distances and TALOS+ dihedral restraints. In total, 8 iterations were performed in ARIA, folding the structure in torsional space, and employing network anchoring for the first 3 iterations. 40 structures were calculated, saving the 15 lowest energy structures for the next round, with the exception of the final iteration where 100 structures were calculated and the 20 lowest energy structures were imported into Analysis. Automatically assigned ambiguous NOEs were manually checked and ambiguity was reintroduced based on a 20 Å distance cut-off.

Following ARIA refinement, an NOE restraint list was generated for structure calculation with Xplor-NIH 2.32 employing the RAMA multi-dimensional torsion angle database potential term^59^. Dihedral and H-bond restraints were also employed (final restraints summarized in Table 1). During iterative Xplor-NIH restraint refinement, NOE restraints were heavily weighted until the structure converged; the weight of dihedral angle restraints was then increased, followed by H-bonds, until the energies began to increase for each class of restraint. Following ensemble calculation with the final set of restraints, water refinement was performed using Xplor-NIH^56^. The 20 lowest energy structures out of 100 calculated in this manner were retained for the final ensemble and visualized using Chimera^60^ and VMD^61^. PROCHECK-NMR^62^, in-house tcl/tk scripts (freely available upon request), and DSSP^63^ were used to assess structure quality, restraint violations and structural features, respectively. The average backbone and all-heavy atom r.m.s.d. values were calculated relative to the lowest energy structure using VMD^59^.

### DOSY-Based Hydrodynamics Measurements

Translational diffusion coefficient (D_C_) values for W_1_, W_2_ and W_3_ proteins (0.2 mM in NMR buffer, 1 mM DSS and 1 mM NaN_3_ at pH 5 (H_2_O:D_2_O = 9:1); 0.06% dioxane as an internal viscosity control^56^) were determined at 303.15 K from ^1^H diffusion ordered spectroscopy (DOSY) experiments employing pulsed field gradient (PFG) NMR^43^ using an 11.7 T Avance NMR spectrometer equipped with a z-axis gradient and a BBFO SmartProbe (Bruker Canada). DOSY (64 scans, sweep width 12 ppm, relaxation delay incorporating presaturation 2 s) employed stimulated echo and longitudinal eddy current delay (LED) with bipolar gradient pulses and two spoil gradients^64^. The envelope of ^1^H signals was attenuated by increasing the gradient strength from 2% to 95% in 16 steps. The observed signal intensity as a function of gradient strength was fit using a single component exponential fit of the signal decay and the D_C_ was determined from the fit using the simfit program within the T_1_/T_2_ Relaxation module of Bruker Topspin 3.1 using the Stejskal-Tanner formula^65^:


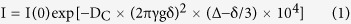


where I is the observed signal intensity, I(0) is the back-calculated unattenuated signal intensity, γ is the gyromagnetic ratio of ^1^H (4257.7 Hz/G), g is the gradient strength (based on maximum amplitude 53.5 G/cm at 100%), δ is the gradient pulse length (8 ms), and Δ is the diffusion time (100 ms). The radius-of-gyration (R_g_, in Å) is calculated as^46^:


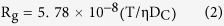


where T is the temperature (in K), η is the viscosity (in cP) estimated using the experimentally observed D_C_ of a dioxane internal standard^45^, and D_C_ is the observed diffusion coefficient (in cm^2^/s). Protein hydrodynamic diameter (d_H_) was inferred from the DOSY-based D_C_ (D_C_^protein^) and corrected for viscosity using the relationship:


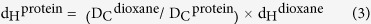


where D_C_^dioxane^ is the measured DOSY-based D_C_ of the dioxane internal standard in a given sample and d_H_^dioxane^ is the known hydrodynamic diameter of dioxane (0.424 nm)^45^.

### DLS-Based Hydrodynamics Measurements

DLS was carried out at an angle of 173° using a 633 nm He-Ne laser using a Zetasizer nano ZS (Malvern, Worcestershire, UK) as described previously^44^. Scattering of filtered (0.45 μm, EMD Millipore) W_1_, W_2_ or W_3_ at both equimolar (~20 μM) and at ~equivalent W-unit concentrations (56 μM W_1_, 28 μM W_2_ and 20 μM W_3_) in NMR buffer was measured (in duplicate) in disposable, 10-mm path length, polystyrene cuvettes (Sarstedt, Montréal, QC, Canada) at 30 °C. Duplicate measurements were made using an automated attenuator 4.65 mm away from the cuvette wall and the resulting autocorrelation function obtained was analysed using Zetasizer software ver. 7.10 under the Protein Analysis Model to determine the average d_H_ of the primary species in each W sample.

### W_2_ and W_3_ Structure Calculation

The W_1_ NOE, H-bond and dihedral angle restraints from the final round of structure calculations (summarized in Table 1) were propagated over residues 200–400 (W_2_ and W_3_) and 400–600 (W_3_) to generate restraint files for W_2_ and W_3_. 100-member structural ensembles were calculated in the same manner as for W_1_ without water refinement using Xplor-NIH 2.32^59^ under the final simulating annealing energy weights used for W_1_ with visualization in Chimera^60^. Direct comparison of the lowest energy 20 ensemble members produced either in the absence of or with DOSY-derived R_g_ restraints (the scaling of R_g_ in the simulated annealing potential^59^, determined iteratively, was the maximum which did not induce a major increase in overall energy) was performed. An in-house python script was used to test for restraint violations. HYDROPRO^66^ was used to calculate the D_C_ of each ensemble member based upon its coordinate file, allowing direct comparison of DOSY derived D_C_ to the ensemble-average behaviour.

### W_2_ Fibre Formation and Mechanical Properties

W_2_ fibres were pulled from 20–200 μM W_2_ protein dissolved either in 50 mM potassium phosphate (pH 7.5) or in NMR buffer, as previously described^48^. Before tensile strength testing, the diameter of each fibre was measured using light microscopy at 400× magnification. Three micrographs were employed for each fibre, two taken near each end and one in the ~middle of the fibre. From each micrograph, three locations were analysed using ImageJ 1.47 v^67^ to determine the diameter of the fibre and the resulting nine diameter estimates for each fibre were averaged. The cross sectional area of each fibre was calculated, assuming that the fibres were circular in cross-section. Tensile strengths of fibres were measured at 22 ± 2 °C and ~40% humidity, using an Agilent T150 UTM, following previously reported procedures^48^.

### Electron and Atomic Force Microscopy

Scanning electron microscopy (SEM) (S-4700, Hitachi, Tokyo, Japan) was used to observe fibre surfaces at 3 kV. Fibres were fixed on conductive adhesive tape glued onto an SEM stub and then coated with gold particles by an SC7620 mini sputter coater (Quorum Technologies, East Sussex, UK) before SEM imaging. For atomic force microscopy (AFM), a ~5 μL drop of a given protein solution in phosphate buffer ([W_2_] ~20 μM) or in NMR buffer ([W_2_] ~200 μM) was deposited onto a clean glass microscope slide. A ~1 cm long fibre was pulled from the solution and placed back onto the slide, then allowed to air-dry at ambient temperature and pressure. Dry fibres were imaged in intermittent contact mode (22 ± 2 °C, at 47 ± 5% relative humidity) using an atomic force microscope (NanoWizard II Ultra, JPK, Berlin, Germany) mounted on an inverted optical microscope (Axio Observer A1, Carl Zeiss Canada, Toronto, Canada). Cantilevers with ~300 kHz resonance frequency and force constant of 40 N/m with a tip height of 17 μm and nominal radius of curvature of <10 nm at the tip were employed (Tap 300-G, Budget Sensors, Sofia, Bulgaria). AFM image files were processed using v3.3.32 of the NanoWizard IP software (JPK).

### Raman Spectromicroscopy of Fibres

W_2_ fibres produced and prepared as described above were fixed onto glass slides by taping two ends and middle section of the fibres for Raman spectromicroscopy. *A. aurantia* spiders (collected in Florida, USA) were farmed in 20 × 50 × 60 cm cages at 58 ± 5% relative humidity (RH) and 24 ± 2 °C, fed four times weekly with small crickets and weekly with 3 droplets of 10% w/v glucose solution. *A. aurantia* wrapping silk fibres were directly reeled by the spiders around small plastic caps for spectromicroscopy. Spectra were obtained at 22.0 ± 0.5 °C and 20 ± 5% RH using a LABRAM 800HR Raman spectrometer (Horiba Jobin Yvon, Villeneuve d’Ascq, France) coupled to a BX30 (Olympus, Richmond Hill, ON, Canada) fixed stage microscope. An Ar^+^ laser (514 nm; 50 mW) was focused through a 100× objective lens onto a given fibre. To obtain information about molecular orientation, four polarized spectra, labelled XX, XZ, ZX and ZZ, were recorded^68^. The first and second letters indicate the polarization of the incident and scattered radiation, respectively, where Z corresponds to the fibre long-axis and X to the perpendicular direction. 2 × 15 sec acquisitions were collected 3–5 times at 5 positions on 3 W_2_ fibres. Reported spectral data are the average of these acquisitions. A similar procedure was applied to natural fibres, with the polarized spectra resulting from the average of ~12 acquisitions. Spectral manipulations were performed using GRAMS/AI 7.0 (ThermoGalactic, Salem, NH). The spectra were baseline-corrected using a cubic function, 5-point smoothed, and averaged for each fibre. The orientation-insensitive spectrum was calculated and the amide I band decomposed to estimate the content of α-helices and β-sheets, as described previously^11^,^69^.

### Titration-Induced Protein Unfolding

To monitor the global folded state, far-UV CD spectra of W_1_ and W_2_ protein samples (9–20 μM in 20 mM sodium acetate buffer) were recorded at 100 nm/min in 0.1 nm intervals from 260 to 195 nm using a J-810 spectropolarimeter (Jasco, Easton, MD, USA) at 22 ± 2 **°**C in 0.1 cm quartz cuvettes (Hellma, Müllheim, Germany). Protein concentration in a given sample was determined by the absorbance at 210 nm (calculated ε-values: ε_W1_ = 270858 M^−1^cm^−1^, ε_W2_ = 543596 M^−1^cm^−1^). Samples were analysed in duplicate, blank corrected, averaged, and converted to mean residue ellipticity [θ]. DPC was titrated using a relative molar ratios to W_1_ or W_2_ (W_n_:DPC 1:0, 1:1, 1:10; 1:50; 1:100 and 1:500). Guanidinium chloride (GdmCl) and urea were titrated as a function of concentration from 0 to 5 M for urea and 0 to 4 M for GdmCl. The fraction folded as a function of denaturant concentration (C), F(C), was calculated (similarly to thermal denaturation^70^) as:


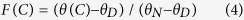


where *θ(C)*, *θ*_*N*_ and *θ*_*D*_ are the ellipticities observed at concentration C, in the native (C = 0) and denatured states (in 5 M urea or 4 M guanidine chloride), respectively.

To monitor localized detergent-induced unfolding by NMR spectroscopy, DPC was added to ^15^N-enriched W_1_ (0.34 mM in NMR buffer) at an increasing molar ratio of W_1_:DPC (10:1, 5:1, 1:1, 1:5, 1:10, 1:50), with ^1^H-^15^N HSQC experiments (8 scans, 2048 × 128 points, recovery delay of 1.5 s) acquired at each titration point using a 16.4 T Bruker Avance III spectrometer equipped with a 5 mm indirect detection TCI cryoprobe at 303.15 K. The change in W_1_ concentration due to volume increase was negligible. 3D backbone NMR experiments (HNCO, HNcaCO, HNCA, HNcoCA, as previously acquired for W_1_^33^) were acquired at the DPC endpoint (0.34 mM W_1_, 20 mM DPC) to facilitate assignment. Perturbation of W_2_ was monitored using an intein-spliced variant with the first W-unit uniformly ^13^C- and ^15^N-enriched and the second W-unit only ^15^N-enriched (0.17 mM in NMR buffer) via the isotopically discriminated (IDIS) ^1^H-^15^N HSQC experiment^71^ at 0 mM and 20 mM DPC (48 scans, 2048 × 128 × 2 points, recovery delay of 1.5 s), allowing simultaneous acquisition of data for both W domains in a single sample. To monitor effects of chaotropes, W_1_ samples (0.34 mM in NMR buffer) were titrated with GdmCl (0, 0.4, 0.6, 0.8, 1, 1.2, and 2 M) and urea (0, 0.5, 1, 1.2, 1.4, 2.0, 2.3, 2.5 M) and monitored using ^1^H-^15^N HSQC experiments (24–32 scans, 2048 × 196 points, recovery delay of 1.5 s) acquired at 11.7 T and 303.15 K on a Bruker Avance spectrometer equipped with a 5 mm BBFO SmartProbe. Concentration variation due to volume change was accounted for to accurately set denaturant concentration at each titration point. W_1_ was diluted over the course of each titration, leading to a final concentration of ~0.25 mM at the GdmCl and urea endpoints. All acquired spectra were processed with NMRPipe^56^ and assigned in CcpNmr Analysis^57^. Combined chemical shift displacement (CSD), weighed by gyromagnetic ratio for ^1^H and ^15^N nuclei^38^, was calculated as a function of amino acid residue between the samples containing no titrant and those containing 20 mM DPC, 0.8 M GdmCl (0.30 mM W_1_), or 2.5 M urea. The spectra chosen for analysis of GdmCl and urea perturbation were not the titration endpoints; rather, these were chosen as the most assignable spectra before denaturation made backbone amide chemical shifts indiscriminable. A control titration using NaCl as the titrant was carried out using a series of 40 μL samples (0, 0.4, 0.6, 0.8, 1, and 1.2 M) though ^1^H-^15^N HSQC experiments (24 scans, 2048 × 96 points, recovery delay of 1.5 s) acquired at 303.15 K on a 16.4 T Bruker Avance III spectrometer equipped with a 1.7 mm TCI probe.

## Additional Information

**Accession codes.** Protein Data Bank (PDB): the atomic coordinates for the 20 lowest energy structures for W_1_ described in this manuscript has been deposited with the following accession code: 2MU3 and appended to previously published NMR assignments (BMRB 17899)^33^. NMR assignments for W_2_ were deposited to the Biological Magnetic Resonance Data Bank (BMRB) by combining chemical shifts of W_2-1_ and W_2-2_ with accession code BMRB 25197. 

**How to cite this article**: Tremblay, M.-L. *et al.* Spider wrapping silk fibre architecture arising from its modular soluble protein precursor. *Sci. Rep.* **5**, 11502; doi: 10.1038/srep11502 (2015).

## Supplementary Material

Supplementary Information

## Figures and Tables

**Table 1:** NMR and refinement statistics for W_1_.

**NMR distance and dihedral constraints**	
Total NOE distance constraints	5241
Intra-residue	1470
Inter-residue	
Sequential (|i-j| = 1)	1368
Medium-range (|i-j| < 4)	908
Long-range (|i-j| > 5)	846
Ambiguous	649
Hydrogen bond distance constraints	25
Total dihedral angle restraints	201
Phi	107
Psi	94
**Structural ensemble statistics**	
Violations (mean and s.d.)	
Distance constraints (Å)	0.07 ± 0.05
Dihedral angle constraints (°)	1.22 ± 1.01
Max. dihedral angle violation (°)	4.38 ± 1.20
Max. distance constraint violation (Å)	0.49 ± 0.16
Deviations from idealized geometry	
Bond lengths (Å)	1.026 ± 0.002
Bond angles (°)	0.302 ± 0.007
Impropers (°)	0.461 ± 0.018
Average pairwise r.m.s.d.^a^(Å)	
Heavy	1.32 ± 0.15
Backbone	0.88 ± 0.18

^a^Pairwise r.m.s.d. for backbone and heavy atom were obtained for the 20 water refined lowest energy structures superposed over the globular core from residues 12-140.

**Table 2:** Mechanical properties for fibres produced using indicated recombinant AcSp1 W_2_ protein relative to those previously reported for W_4_^48^ and native *A. trifasciata*^8^ fibres.

	**W_2_**		**W_4_**	**Native AcSp1**
	**Non-spliced**	**Spliced**		
Breaking strength (MPa)	66.7 ± 15.8	67.8 ± 9.8	115 ± 24	687 ± 56
Breaking strain	0.31 ± 0.11	0.27 ± 0.01	0.37 ± 0.11	0.86 ± 0.03
Toughness (J·cm^-3^)	18.4 ± 10.4	16.5 ± 6.92	33.8 ± 13.5	376 ± 39
Young’s modulus (GPa)	1.69 ± 0.68	1.48 ± 0.68	2.44 ± 0.54	~10

**Table 3:** Comparison of secondary structure for recombinant vs. native AcSp1 dope and fibres.

	**Dope**^a^		**Fibre**^a^		
	**W_2_**	***N. clavipes***	**W_2_**	***A. aurantia***	***N. clavipes***
α-helix (%)	40	49	32	33	24
β-sheet (%)	0	0	28	27	30

^a^Proportions in W_2_ in dope are based upon the ensemble of W_1_ structures determined in NMR buffer, given that W_2_ in NMR buffer is fibre-forming-competent. All other proportions are based upon amide I band orientation-insensitive Raman spectral decomposition. The uncertainty on the values is ±3% based on the reproducibility of the experiments and curve-fitting procedure^11^. *N. clavipes* results were previously published^7^ and are provided for comparison. Decomposition of W_2_ and *A. aurantia* fibres is detailed in Supplementary Fig. S7.
