# Supplementary material for: Spider wrapping silk fibre architecture arising from its modular soluble protein precursor
Source: Sci Rep. 2015 Jun 26;5:11502. doi: 10.1038/srep11502 (PMC4481645; doi:10.1038/srep11502)
Supplement: Supplementary Information [file srep11502-s1.pdf]

# Supplementary information for:

# Spider wrapping silk fibre architecture arising from its modular soluble protein precursor

Marie-Laurence Tremblay[1†], Lingling Xu[1,2†], Thierry Lefèvre[3], Muzaddid Sarker[1], Kathleen E. Orrell[1], Jérémie Leclerc[3], Qing Meng[2], Michel Pézolet[3], Michèle Auger[3], Xiang-Qin Liu[1]* & Jan K. Rainey[1,4]*

[1] Department of Biochemistry & Molecular Biology, Dalhousie University, Halifax, NS, Canada.

[2] Institute of Biological Sciences and Biotechnology, Donghua University, Shanghai, P.R. China.

[3] Département de Chimie, Regroupement québécois de recherche sur la fonction, la structure et l'ingénierie des protéines (PROTEO), Centre de recherche sur les matériaux avancés (CERMA) Université Laval, Québec, QC, Canada.

[4] Department of Chemistry, Dalhousie University, Halifax, NS, Canada.

Notes:

[†] These authors contributed equally

* Address correspondence to: paul.liu@dal.ca or jan.rainey@dal.ca

**Table of Content**

Supplementary Figs. S1-S12

Supplementary Table S1

References

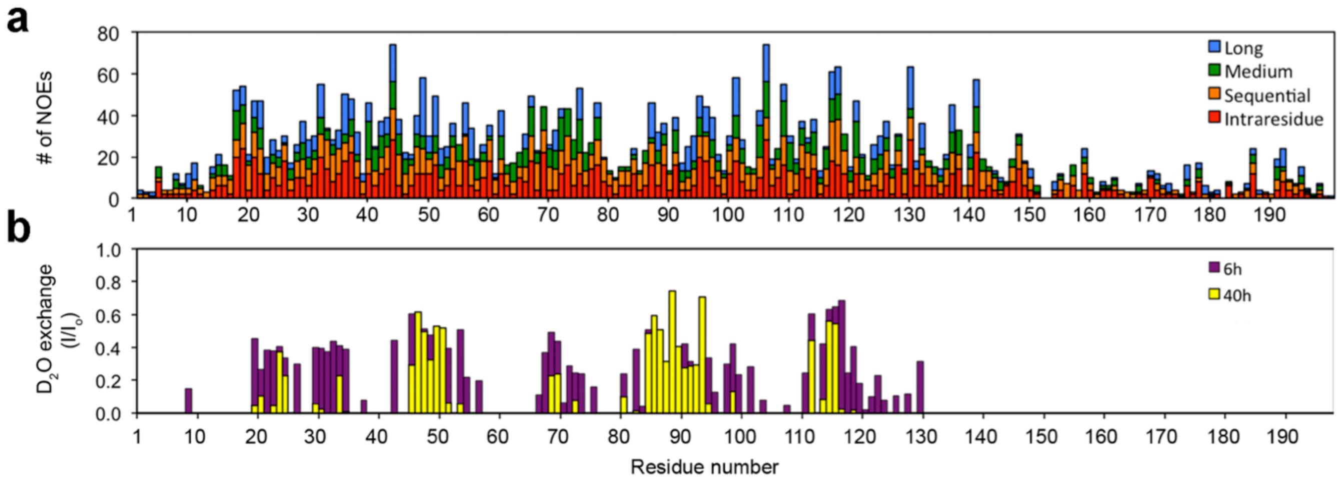


**Supplementary Fig. S1. Graphical summary of NOE, TALOS+ and H-bond restraints.** (a) Graphical representation of the distribution of unambiguous NOE restraints as a function of residue following restraint refinement (*i.e.*, those employed in the final round of structure calculation using Xplor-NIH[1] with water refinement). Definitions employed are sequential: i ± 1; medium: i ± 2, 3, or 4; and, long: i ± > 4. (b) $^{1}H$-$^{15}N$ HSQC peak volume ratio between 0 h ($I_o$) and 6 or 40 h ($I$) remaining after exchange of $H_2O$ with $D_2O$. The ratios are cumulative.

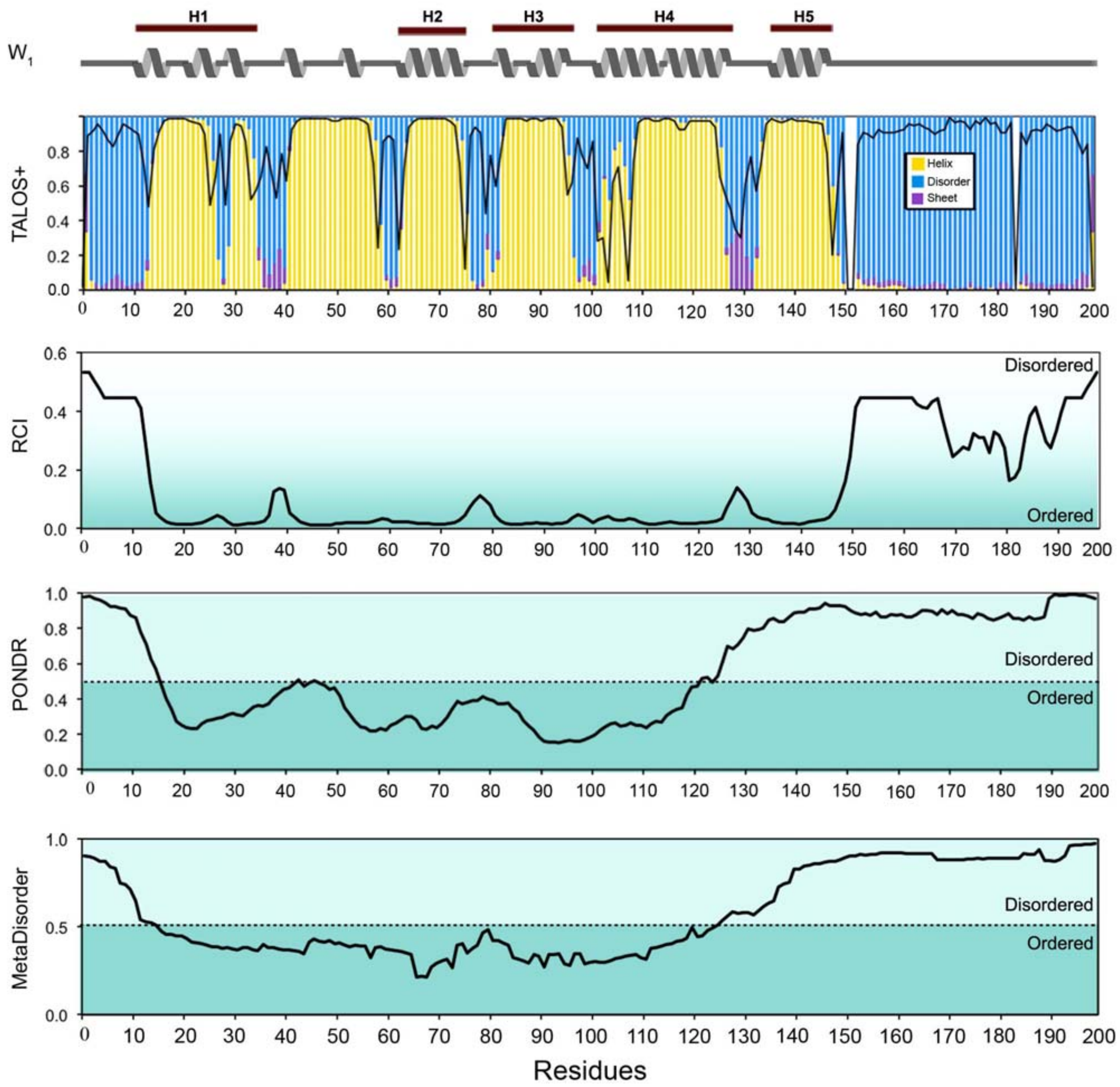


**Supplementary Fig. S2. Chemical shift and sequence-based prediction of intrinsic disorder in $W_1$.** Predictions of structured vs. disordered segments in $W_1$ are illustrated based upon chemical shift (TALOS+[2] and the random coil index (RCI)[3]) and amino acid sequence (PONDR[4] and MetaDisorder[5]). An extended cartoon representation of the converged features of the calculated $W_1$ structural ensemble is overlaid, with extended helical segments denoted. The black line in the TALOS+ panel indicates the confidence of a given secondary structure assessment. The prediction of disorder/order by RCI, PONDR, and MetaDisorder are presented as a gradient (RCI) or a structural threshold (PONDR and MetaDisorder).

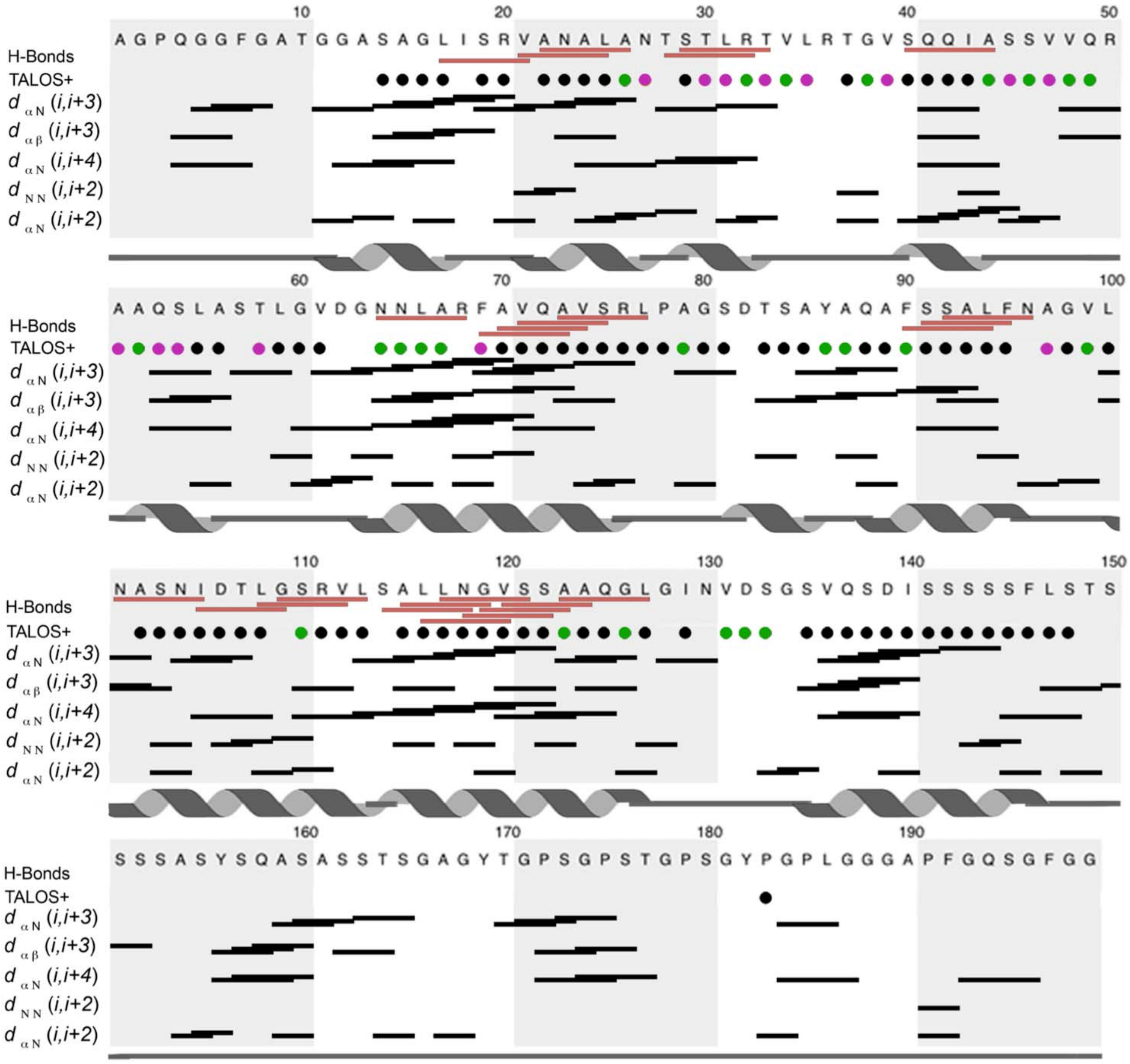


**Supplementary Fig. S3. Graphical summary of restraints retained in final $W_1$ structural calculations.** H-bond, TALOS+ dihedral angle restraints[2] (circles coloured black if both ϕ and ψ are restrained for the given residue, magenta for ϕ only and green ψ for only) and canonical[6] helical i+2, 3 and 4 NOE restraints are depicted following restraint refinement. A linear cartoon structure of $W_1$ is displayed at the bottom, on the basis of DSSP[7] analysis of the final 20-member NMR ensemble.

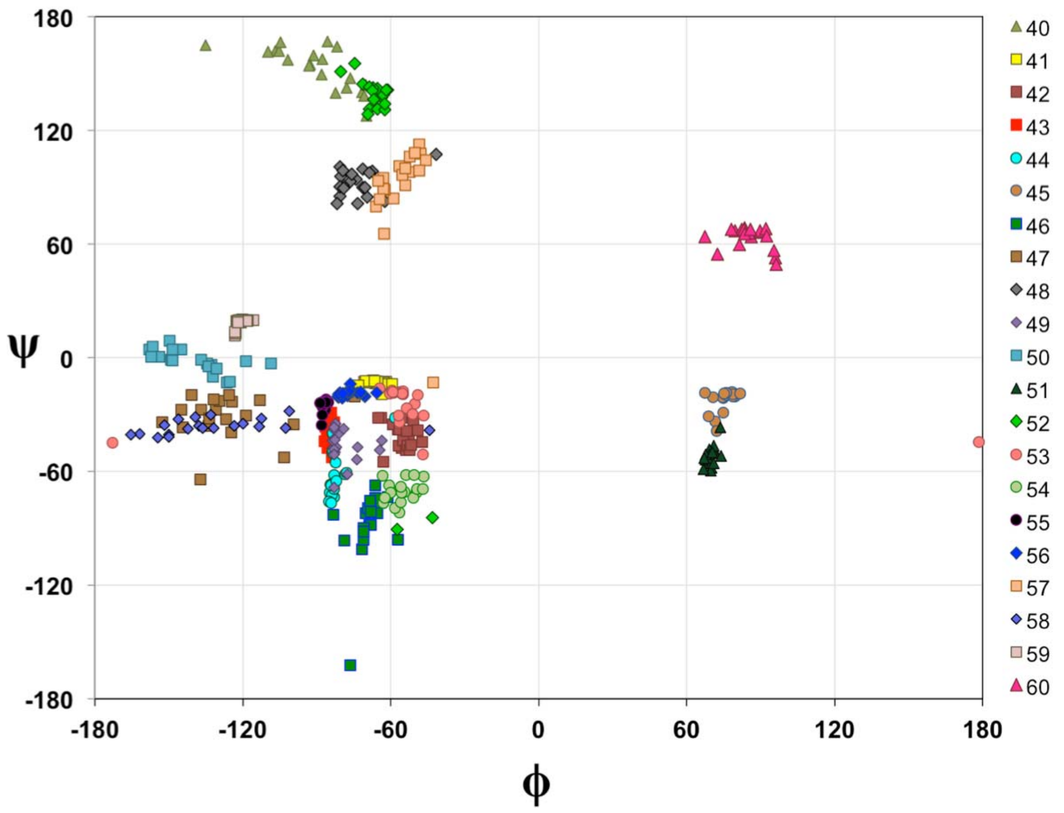


**Supplementary Fig. S4. Ramachandran plot by amino acid over putative helix 2 region of chemical shift-based topology[8].** Clearly, 13 of the 20 amino acids over residues 40-60 exhibit α-helical ϕ and ψ dihedral angle pairs. The helical runs are interrupted by residues 45S, 51A and 60G, located in the glycine region or the turn II region, and residues 40Q, 48V, 52A and 57S, in the β-strand region.

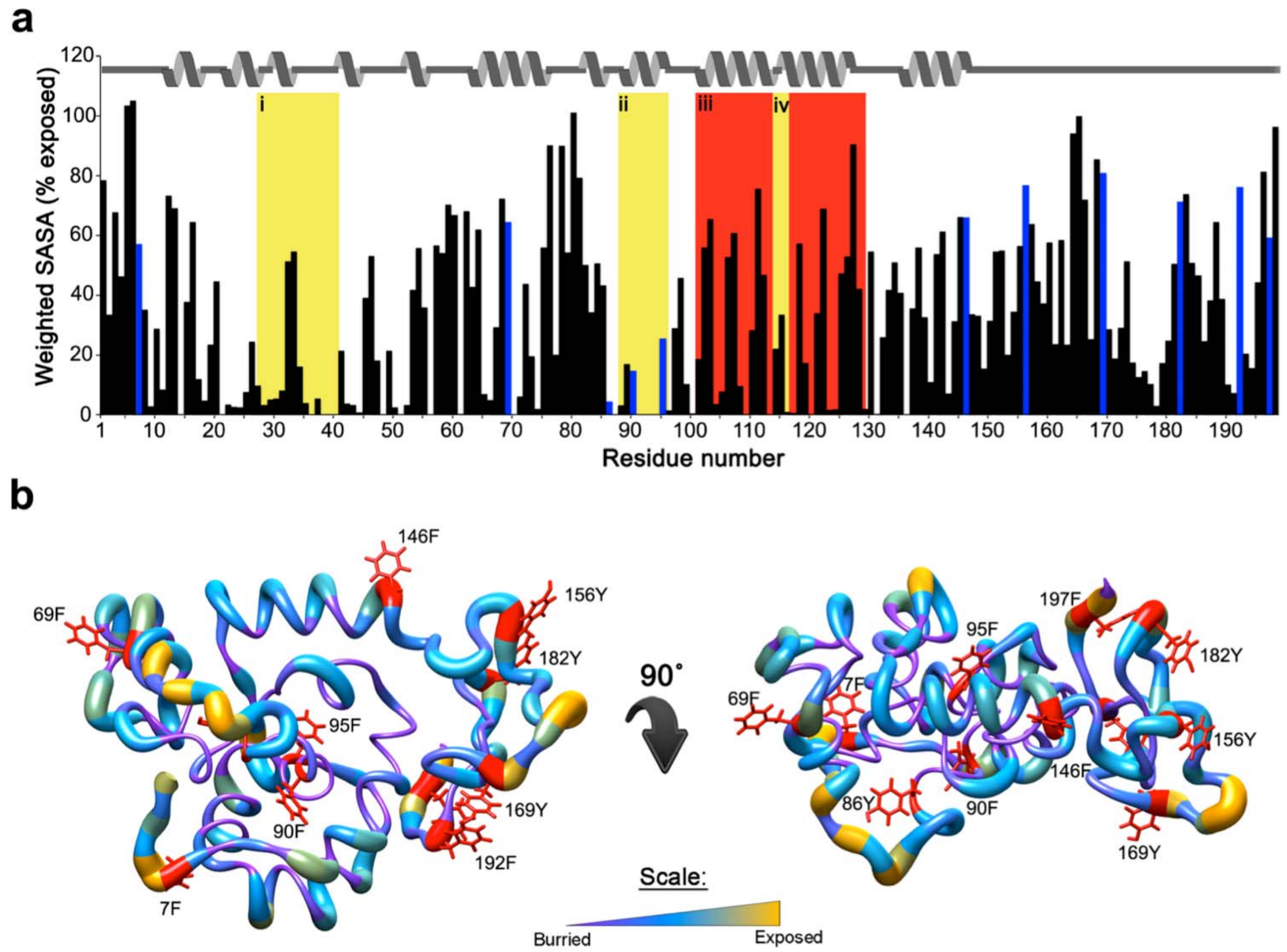


**Supplementary Fig. S5. Accessible surface area as a function of position in $W_1$.** The solvent accessible surface area (SASA) was calculated as a function of residue for each ensemble member using DSSP[7], weighted with respect to the total surface area for each residue type reported by Yuan *et al.*[9] to provide relative SASA values (as a percentage). (a) Graphical representation of SASA per residue. In blue are the aromatic residues that fall in the top 50 most exposed residues. The highlighted blocks are as follows: "i" (yellow) is the region most perturbed by titration with DPC, urea, and GdmCl (Supplementary Fig. 8); "ii" (yellow) is the central helical region where 90F and 95F are solvent exposed in opposing directions; in "iii" (red) is the amphipathic helix 5 which displays a kink at "iv" (yellow). (b) Lowest energy $W_1$ ensemble member coloured and scaled by thickness according to weighted DSSP SASA values. Aromatic residues are coloured red.

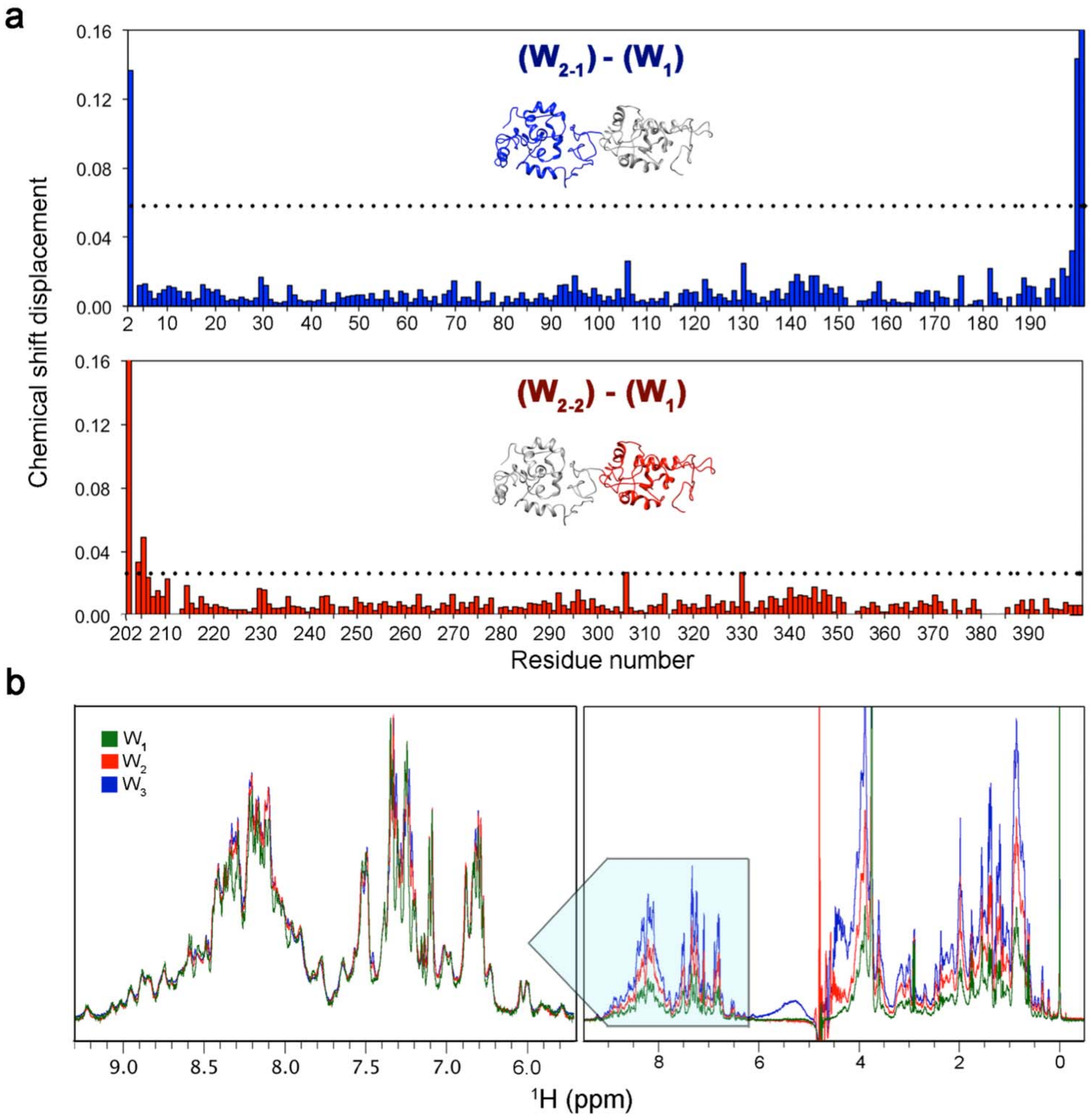


**Supplementary Fig. S6. Comparison of NMR chemical shifts for $W_1$, $W_2$ and $W_3$.** (a) Quantitative combined chemical shift displacement (CSD) comparison[10] between $W_1$ and $W_2$ ($W_{2\text{-}1}$-$W_1$ in blue; $W_{2\text{-}2}$-$W_1$ in red, where n in $W_{2\text{-}n}$ refers to the W unit that is uniformly $^{15}N$ and $^{13}C$-enriched in a given segmentally-labelled $W_2$ concatemer). The combined CSD for $C^{\alpha}$, $H^N$, N, and CO was calculated using the square root of the sum of squares weighted by gyromagnetic ratio. The mean plus one standard deviation ($W_{2\text{-}1}$: 0.054 + 0.011, $W_{2\text{-}2}$: 0.017 + 0.008) of each CSD is represented by the dotted line, providing an estimated significance in terms of CSD. (b) Overlay of the 1D $^1$H-NMR spectra for equimolar samples of indicated $W_n$ sample. The intensity of the full spectral overlay on the right is as observed, while the zoom of the amide region on the left is normalized.

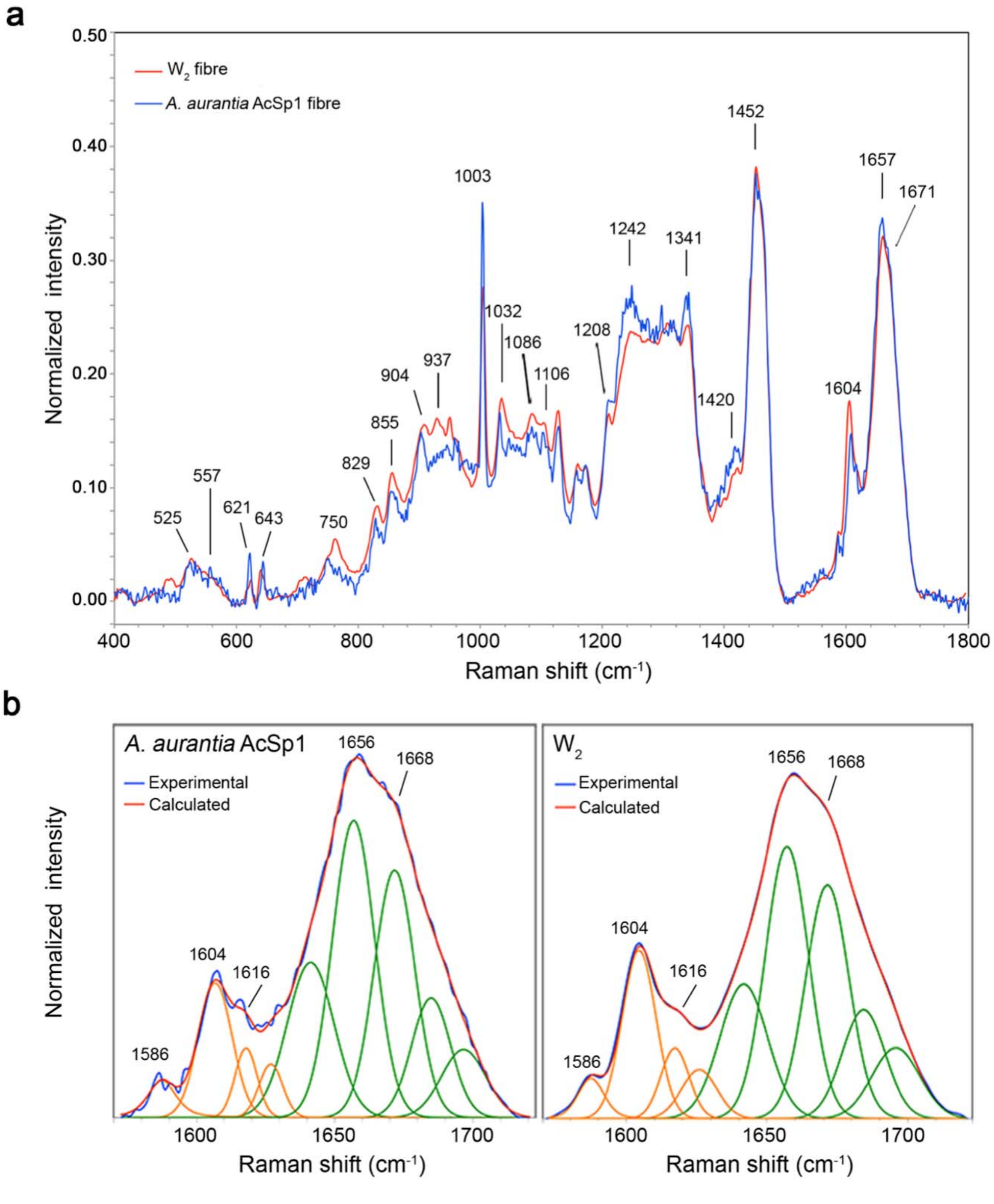


**Supplementary Fig. S7. Protein secondary structure comparison of native *Argiope aurantia* aciniform silk fibre to fibres drawn from $W_2$ solution.** (a) Overlaid orientation-insensitive Raman spectra[11] averaged over multiple fibres/positions (detailed in Methods) of the indicated type of aciniform silk fibre. (b) Spectral decomposition[12] of the amide I band for the indicated fibre type. The amide I decomposition component at 1656 cm$^{-1}$ was assigned to α-helices and that at 1668 cm$^{-1}$ to β-sheets. The α-helix and β-sheet content were calculated from the ratio of the area of the given band to the total amide I band area.

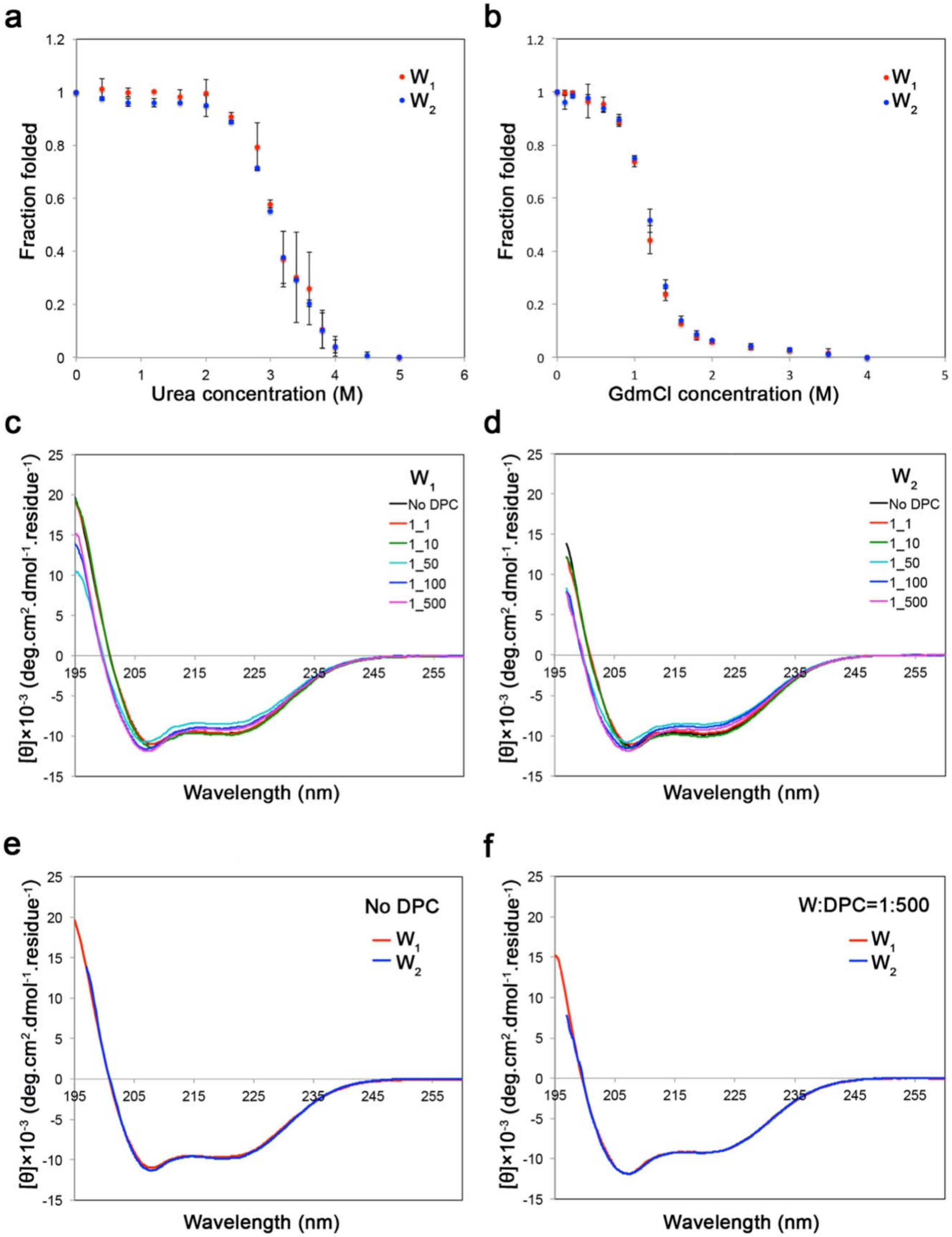


**Supplementary Fig. S8. Far-UV CD spectroscopy comparing effects of chaotrope and detergent addition to $W_1$ and $W_2$.** (a) Urea and (b) guanidinium chloride (GdmCl) titration-based denaturation curves, with fraction folded determined using equation 4 by ellipticity for the characteristic 222 nm $\alpha$-helical band at $22\pm2$˚C. Titration of $W_1$ (c) and $W_2$ (d) with DPC at indicated molar ratio alongside overlay of titration start (e) and end (f) points for $W_1$ and $W_2$.

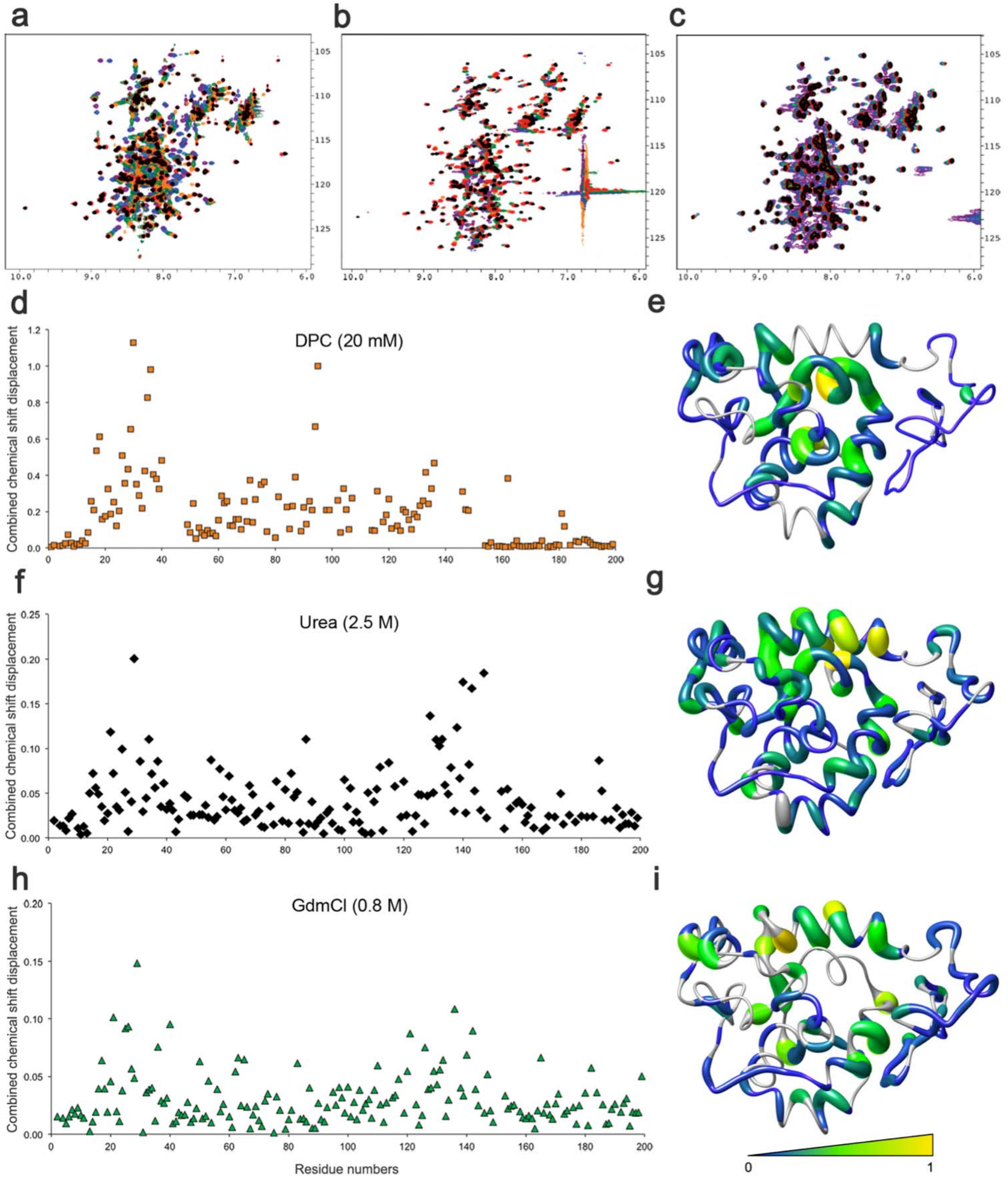


**Supplementary Fig. S9. Denaturation of $W_1$ followed by NMR.** (a-c) $^{1}H$-$^{15}N$ HSQC spectra of representative titration points for $W_1$ with (a) DPC (black 0.0 mM, red 0.07 mM, orange 0.34 mM, green 2.70 mM, light blue 4.70 mM, blue 10.0 mM, purple 20.0 mM); (b) GdmCl (black 0.0 M, red 0.4 M, orange 0.6 M, green 0.8 M, blue 1.0 M, purple 1.2 M); and, (c) urea (black 0.0 M, red 0.5 M, green 1.0 M, blue 2.0 M, purple 2.5 M). Quantitative chemical shift displacements (CSDs) are presented for indicated perturbant relative to unperturbed (d, f, h) using the combined CSD method weighted by gyromagnetic ratio[10]. These CSD results are represented in a normalized manner on the lowest energy $W_1$

structure from the 20-member ensemble using colour and cartoon thickness (e, g and i, corresponding to d, f and h, respectively; residues coloured grey were overlapped or not possible to follow through the titration to the indicated perturbant concentration.)

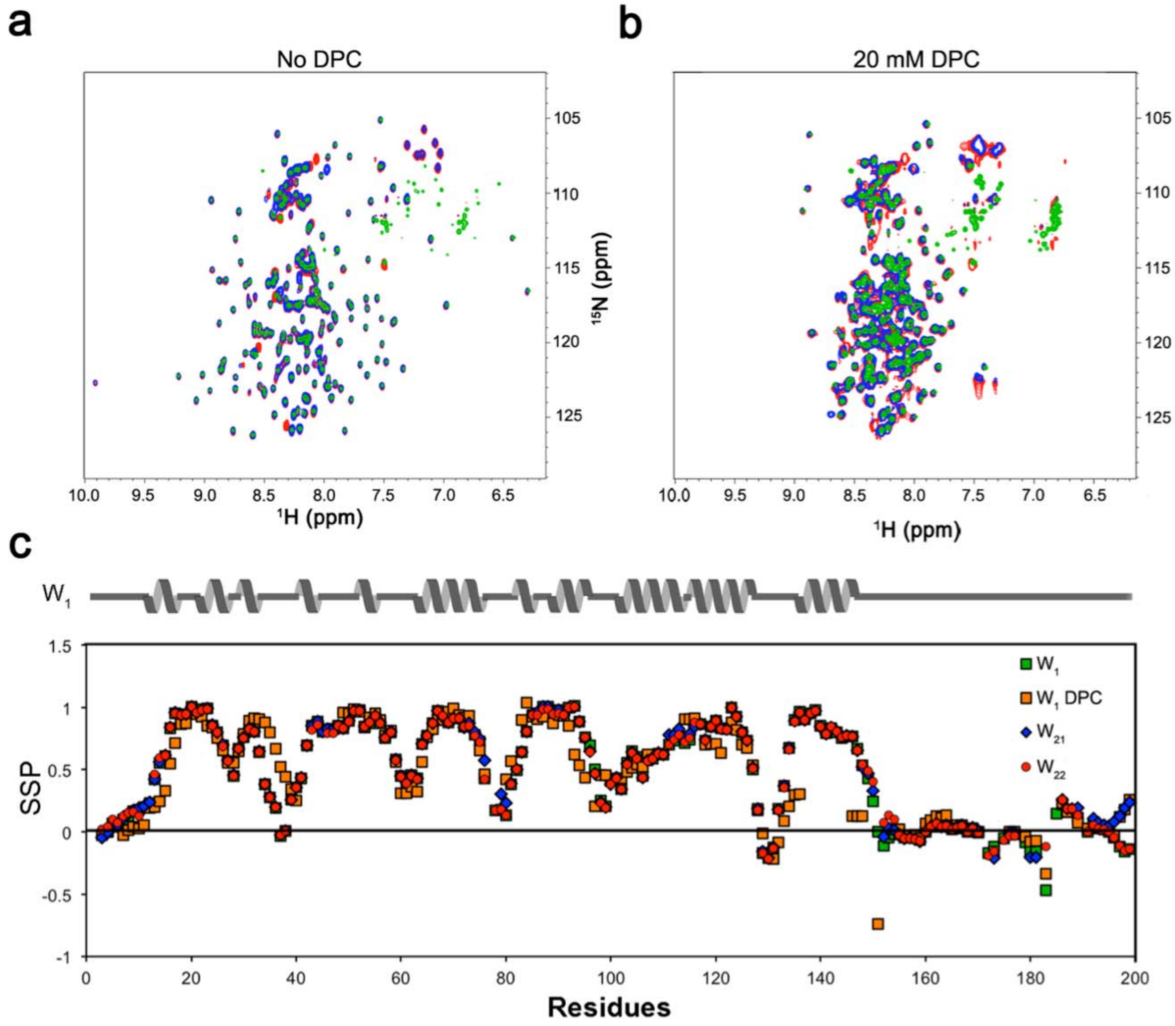


**Supplementary Fig. S10. Perturbation of $W_2$ by detergent DPC.** (a-b) Isotopically discriminated (IDIS) $^{1}$H-$^{15}$N HSQC experiments[13] at given DPC concentration acquired for an intein-spliced $W_2$ constructed such that the first W in $W_2$ (blue, $W_{2\text{-}1}$) is uniformly $^{15}$N and $^{13}$C-enriched while the second W is only $^{15}$N-enriched (red, $W_{2\text{-}2}$). A standard $^{1}$H-$^{15}$N HSQC for $W_1$ is overlaid in green. The only peaks that differ between $W_1$ and each $W_2$ are those at the junction between the two W units. (c) Secondary structure propensity (SSP[14]) analysis for $W_1$, $W_{2\text{-}1}$, and $W_{2\text{-}2}$ using HN, CO,CA, and N chemical shifts in standard NMR conditions and for $W_1$ at the DPC endpoint ($W_1$_DPC legend entry). An extended cartoon representation of the structural features of the $W_1$ structural ensemble is overlaid above the SSP plot.

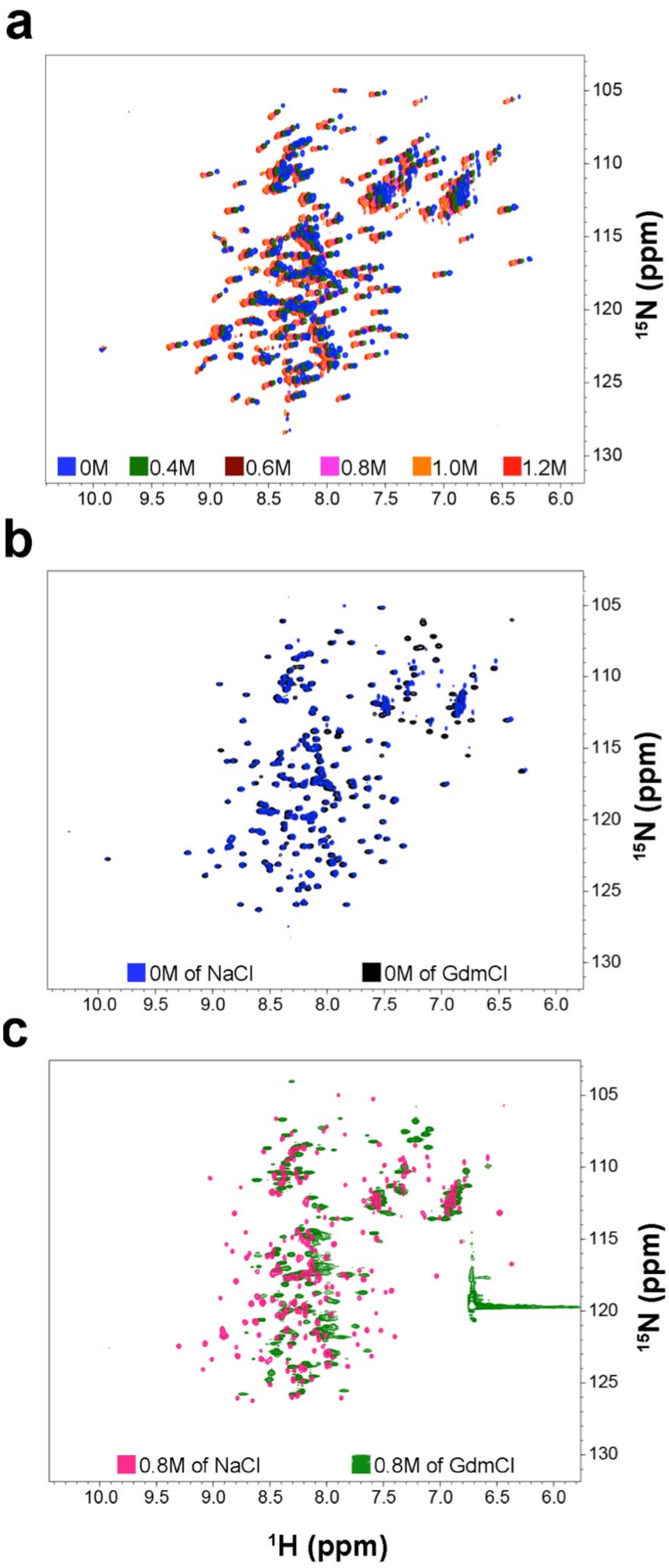


**Supplementary Fig. S11. Effect of ionic strength on $W_1$ followed by NMR.** (a) $^{1}$H-$^{15}$N HSQC spectra of representative titration points for $W_1$ with NaCl. (b) Overlay of the NMR spectrum of $W_1$ before the titration. (c) Overlay of $W_1$ spectra in either 0.8M NaCl or 0.8M GdmCl.

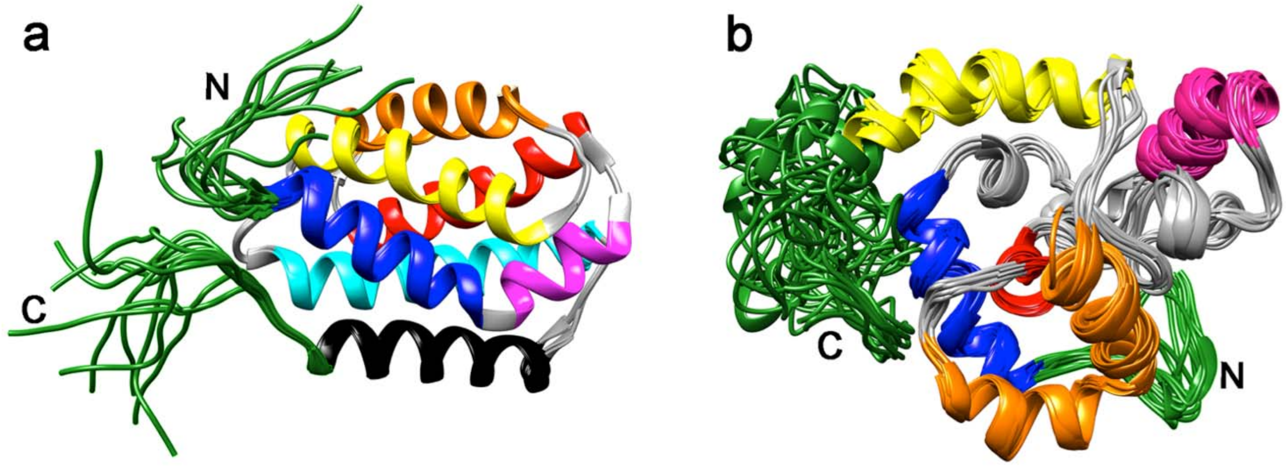


**Supplementary Fig. S12. Comparison of (a) previously reported truncated *N. antipodiana* AcSp1[15] and (b) $W_1$ structure.** In each protein, helices 1-5 are coloured consistently: 1 – blue; 2 magenta; 3 – red; 4 – orange; 5 – yellow. Helices 6 and 7 of the *N. antipodiana* AcSp1 unit are coloured cyan and black, respectively. Inter-helical segments are coloured grey, while the N- and C-termini are coloured green. For consistency, superpositions of 10 conformers are shown for each AcSp1 protein: (a) using all conformers from PDB entry 2LYI; (b) using the lowest energy 10 conformers in the $W_1$ structure reported herein.

**Supplementary Table S1.** Experimentally observed $D_C$ for $W_1$, $W_2$ and $W_3$ from PFG-DOSY NMR ($D_C^{DOSY}$) with corresponding $d_H$ ($d_H^{DOSY}$) and $R_g$ estimate; $d_H$ measured by dynamic light scattering ($d_H^{DLS}$); $D_C$ calculated on the basis of structural ensemble determined under given conditions ($D_C^{Structure}$); and, illustration of lowest energy $W_1$ NMR ensemble member or representative members (3 of the 20 lowest energy ensemble members closest to $D_C^{DOSY}$) of given $W_2$ or $W_3$ ensemble.

| Protein | $D_C^{DOSY}$ ($\times 10^{-10}$ m$^2$/s)[a] | $d_H^{DOSY}$ (nm)[b] | $d_H^{DLS}$ (nm)[c] | $R_g$ (Å)[d] | $D_C^{Structure}$ ($\times 10^{-10}$ m$^2$/s)[e] | |
|---|---|---|---|---|---|---|
| | | | | | Without $R_g$ | With $R_g$ |
| $W_1$ | 0.983±0.012 | 4.3±0.06 | N/A[f] | 17.1 | 0.926±0.006 (6%) 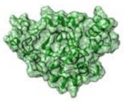 | 0.915±0.006 (7%) 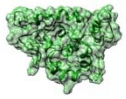 |
| $W_2$ | 0.737±0.015 | 5.7±0.1 | 5.6±0.5 | 22.5 | 0.648±0.010 (12%) 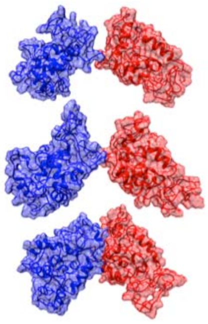 | 0.697±0.005 (5%) 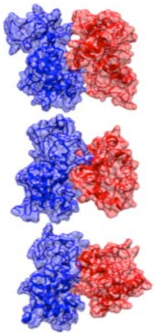 |
| $W_3$ | 0.577±0.004 | 7.3±0.05 | 6.3±0.3 | 28.6 | 0.546±0.009 (5%) 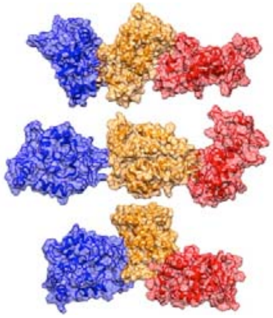 | 0.579±0.004 (0%) 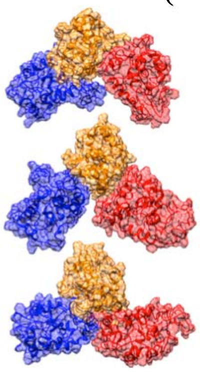 |

[a] Average ± average deviation obtained from 4 protein peaks (2 experiments) measured using equation 1[16].

[b] Average ± average deviation for 4 protein peaks (2 experiments) based on $D_C^{DOSY}$ corrected for viscosity using internal dioxane standard using equation 3[17].

[c] Average ± average deviation of two replicates for two independent samples.

[d] Estimated using equation 2[18].

[e] Average HYDROPRO[19]-derived $D_C$ over a given 20-member atomic-level structural ensemble determined using the solution NMR restraints both without and with $R_g$. The error reported is the average deviation of $D_C$ over a given ensemble; the bracketed percentage is the deviation from $D_C^{DOSY}$.

[f] Not determinable due to scattering from nanoparticle (micelle) population in solution, consistent with previous observation[20].

## REFERENCES


1. Schwieters, C. D., Kuszewski, J. J. & Clore, G. M. Using Xplor–NIH for NMR molecular structure determination. *Prog. Nucl. Mag. Res. Sp.* **48,** 47–62 (2006).
2. Shen, Y., Delaglio, F., Cornilescu, G. & Bax, A. *J. Biomol. NMR.* **44,** (2009).
3. Berjanskii, M. V. & Wishart, D. S. The RCI server: rapid and accurate calculation of protein flexibility using chemical shifts. *Nucleic Acids Res* **35,** 7 (2007).
4. Xue, B., Dunbrack, R. L., Williams, R. W., Dunker, A. K. & Uversky, V. N. PONDR-FIT: a meta-predictor of intrinsically disordered amino acids. *Biochim. Biophys. Acta* **1804,** 996–1010 (2010).
5. Kozlowski, L. P. & Bujnicki, J. M. MetaDisorder: a meta-server for the prediction of intrinsic disorder in proteins. *BMC Bioinf.* **13,** 111 (2012).
6. Wüthrich, K. *NMR of Proteins and Nucleic Acids*. (Wiley-Interscience, 1986).
7. Kabsch, W. & Sander, C. Dictionary of protein secondary structure: Pattern recognition of hydrogen-bonded and geometrical features. *Biopolymers* **22,** 2577 (1983).
8. Xu, L., Tremblay, M.-L., Meng, Q., Liu, X.-Q. & Rainey, J. K. 1H, 13C and 15N NMR assignments of the aciniform spidroin (AcSp1) repetitive domain of Argiope trifasciata wrapping silk. *Biomol NMR Assign* **6,** 147–151 (2012).
9. Yuan, Z., Zhang, F., Davis, M. J., Bodén, M. & Teasdale, R. D. Predicting the solvent accessibility of transmembrane residues from protein sequence. *J. Proteome Res.* **5,** 1063–1070 (2006).
10. Schumann, F. H. *et al.* Combined chemical shift changes and amino acid specific chemical shift mapping of protein-protein interactions. *J. Biomol. NMR.* **39,** 275–289 (2007).
11. Lefèvre, T., Rousseau, M.-E. & Pézolet, M. Orientation-insensitive spectra for Raman microspectroscopy. *Appl Spectrosc* **60,** 841–846 (2006).
12. Lefèvre, T., Rousseau, M.-E. & Pézolet, M. Protein secondary structure and orientation in silk as revealed by Raman spectromicroscopy. *Biophys. J.* **92,** 2885–2895 (2007).
13. Bermel, W., Tkach, E. N., Sobol, A. G. & Golovanov, A. P. Simultaneous measurement of residual dipolar couplings for proteins in complex using the isotopically discriminated NMR approach. *J. Am. Chem. Soc.* **131,** 8564–8570 (2009).
14. Marsh, J. A., Singh, V. K., Jia, Z. & Forman-Kay, J. D. Sensitivity of secondary structure propensities to sequence differences between α- and γ-synuclein: implications for fibrillation. *Protein Sci.* **15,** 2795–2804 (2006).
15. Wang, S., Huang, W. & Yang, D. NMR structure note: repetitive domain of aciniform spidroin 1 from Nephila antipodiana. *J. Biomol. NMR.* **54,** 415–420 (2012).
16. Stejskal, E. O. & Tanner, J. E. Spin diffusion measurements: spin echoes in the presence of a time-dependent field gradient. *J. Chem. Phys.* **42,** 288 (1965).
17. Jones, J. A., Wilkins, D. K., Smith, L. J. & Dobson, C. M. Characterisation of protein unfolding by NMR diffusion measurements. *J. Biomol. NMR.* **10,** 199–203 (1997).
18. Tyn, M. T. & Gusek, T. W. Prediction of diffusion coefficients of proteins. *Biotechnol. Bioeng.* **35,** 327–338 (1990).
19. Ortega, A., Amorós, D. & García de la Torre, J. Prediction of hydrodynamic and other solution properties of rigid proteins from atomic- and residue-level models. *Biophys. J.* **101,** 892–898 (2011).
20. Xu, L. *et al.* Nanoparticle self-assembly by a highly stable recombinant spider wrapping silk protein subunit. *FEBS Lett* **587,** 3273–3280 (2013).
